# Effects of different cultivation media on root bacterial community characteristics of greenhouse tomatoes

**DOI:** 10.3389/fmicb.2023.1182347

**Published:** 2023-05-18

**Authors:** Xinjian Zhang, Qiang Li, Fangyuan Zhou, Susu Fan, Xiaoyan Zhao, Chi Zhang, Kun Yan, Xiaoqing Wu

**Affiliations:** ^1^Shandong Provincial Key Laboratory of Applied Microbiology, Ecology Institute, Qilu University of Technology (Shandong Academy of Sciences), Ji’nan, China; ^2^School of Agriculture, Ludong University, Yantai, China

**Keywords:** greenhouse tomato, root bacterial community, substrate, soil, transplantation

## Abstract

Tomato, as a typical greenhouse crop, is commonly first planted as seedlings in a variety of substrates before being transplanted into soil. However, there is rare research on the characteristics of the bacterial community in tomato roots under this planting mode. In this study, tomatoes were planted in pots containing three different cultivation media, including soil and two types of substrates in a greenhouse, followed by a transplanting treatment. After collecting tomato root samples, high-throughput sequencing and bioinformatic analysis were used to compare the differences in bacterial diversity and functions between tomato roots before and after transplanting in different cultivation media. In total, 702776 sequences were obtained, and the OTUs were belonging to 109 genera, 58 families, 41 orders, 14 classes, and 12 phyla. Among the three cultivation media, the *β*-diversity was significant, and there was a slight difference in bacterial species diversity along with a large difference in their abundance at the genus level. Soil and both substrates had 79 bacterial genera in common, these genera accounted for 68.70%, 76.70%, and 71.17% of the total genera found in the soil, substrate 1, and substrate 2, respectively. After being transplanted from the two substrates to the soil, the bacterial community structure and abundance exhibited similarities with those found in the soil. Furthermore, based on microbial function prediction, the microbial communities in the two-substrate environment demonstrated a greater potential for promoting growth, while the microbial communities in the soil exhibited a greater tendency to exert their antibacterial potential. Our findings offer theoretical support for the creation of artificially reconstructed microbial communities in greenhouse cultivation.

## 1. Introduction

Increasing market demand and quality requirements have gradually switched the cultivation of cash crops, such as vegetables and fruits, from open-field planting to facility production mode. Tomato is an important cash crop in greenhouse agriculture in China. Its planting scale is among the top worldwide, with a remarkable economic benefit (Ren et al., 2022). Tomato planting in a greenhouse is mainly divided into two stages: seedling in a seedling substrate and transplantation to soil for plant growth (Martínez-Gutiérrez et al., 2016). The seedling substrate is generally composed of nutrients (such as natural exploitation of peat and straw fermentation) and accessories (such as perlite, vermiculite, and coconut bran), with light texture, good water, and fertilizer retention, and air permeability. Germination rate and yield remarkably increase before and after seedling transplantation compared with direct soil seeding (Martínez-Gutiérrez et al., 2016). However, continuous cropping obstacle has arisen in the high-intensity cultivation of greenhouse tomato (Zheng et al., 2020). Introducing microorganisms in greenhouse tomato planting can improve the availability of mineral elements, regulate secondary metabolite production in the rhizosphere, and enhance crop resistance to plant diseases and insect pests (Almaghrabi et al., 2013; Ge et al., 2015; Ricci et al., 2019; Zheng et al., 2020). However, only the unilateral effect of functional bacterial strains has been previously considered for selecting the externally added microorganisms. The selection of optimal plant genotypes for microorganism colonization is relatively ignored, leading to the difficult colonization of bacterial strains in crops and their short-term and unstable effects in practical application.

With the advancement of high-throughput sequencing technology, researchers have found that plants are enriched with various microorganisms both on the surface and inside. These microorganisms encode more genes than the host plants and form a stable community structure through collaboration and competition, which is crucial for crop disease and adversity resistance, growth, and development (Trivedi et al., 2020; Compant et al., 2021; Bai et al., 2022). Rhizosphere engineering helps improve plant growth, disease resistance, and stress resistance by artificially reconstructing the plant root community and has emerged to solve the continuous cropping obstacle of plants and crops (Ahkami et al., 2017; Hakim et al., 2021). However, no reports have been found that focus on the changes in crop root colonization microorganisms’ community characteristics in the substrate-to-soil transplanting planting mode. Thus, further research in the above field is required to support rhizosphere engineering technology in the transplanting mode in greenhouse agriculture. Previously, we demonstrated the bacterial community characteristics of a typical facility crop cucumber in the substrate and soil (Zhou et al., 2022). In this study, we compared the differences in the root bacterial community of another typical greenhouse crop tomato in the substrate and soil cultivation. We investigated the effects of substrate transplantation on the soil on bacterial community diversity. This study will help provide data to support the establishment of rhizosphere engineering technology in greenhouse agriculture.

## 2. Materials and methods

### 2.1. Experimental materials

The tomato variety Helan Yingfen was used. The cultivation medium comprised cinnamon soil collected from the experimental base of Shandong Shangdao Biotechnology Co., Ltd. (117°58′58″ E; 36°34′41” N), tomato growing greenhouse soil, Substrate 1 was the seedling cultivation substrate produced by Shandong Tianjiaoyuan Biotechnology Co., Ltd., and comprised a mixture of semi-decomposed straw/ cow dung (volume ratio = 3:1) with perlite, vermiculite, and coir. Substrate 2 was the seedling cultivation substrate produced by Hunan Xianghui Agricultural E-commerce Co., Ltd., and comprised peat with designed coir, vermiculite, and perlite.

### 2.2. Pot experiment of tomato seedlings in different cultivation media

The experiment was conducted in mid-November 2020 in the same tomato-planting greenhouse at the experimental base of Shandong Shangdao Biotechnology Co., Ltd. Soil and two substrates were evenly filled in 10 and 20 planting pots, respectively, with a diameter of 25 cm and a depth of 20 cm. Three tomato seeds were planted in each pot. After conventional watering management, the pots with different cultivation media were placed in the center of the greenhouse in a cross arrangement. When a plant germinated and reached the 1–2 leaf stage, the tomato seedlings were maintained at the same growth in each pot and routinely managed until they grew to 6–7 leaves. Then, 20 pots were filled with soil, and 10 tomato seedlings planted in the two substrates were randomly divided, transplanted into the 10 pots of soil planting pots, and transferred into the greenhouse for continuous cultivation. The treatments were as follows: soil cultivation (S), substrate 1 cultivation (M1), substrate 2 cultivation (M2), substrate 1 transplanted to soil cultivation (M1_to_S), and substrate 2 transplanted to soil cultivation (M2_to_S). Tomatoes were grown to the flowering stage for later use.

### 2.3. Preparation of tomato root sequencing samples

At the mid-flowering stage of tomato, six uniformly growing tomato plants were selected from each treatment as the six biological replicate samples for subsequent sequencing. The aboveground parts were cut, the roots were completely removed after disassembling the pot, and the cultivation media attached to the roots were eliminated. The roots were cut into small pieces using sterile scissors, and 10 g was transferred to a sterile 50 mL centrifuge tube and then placed on ice. Before this process, scissors and other metal tools were sterilizer with fire and rinsed with sterile water thrice to avoid cross-contamination between samples. After processing and sampling, the sample tubes were transported to the laboratory in an ice box.

A pre-configured 1× phosphate-buffered saline (PBS) supplemented with 8 g NaCl, 0.2 g KCl, 1.44 g Na_2_HPO_4_, and 0.24 g KH_2_PO_4_ to 1 L, pH 7.4, and was sterilized at 121°C for 20 min and then cooled it, and set aside. After 35 mL of 1 × PBS was added to each sample tube, it was shaken and washed on a shaking table at normal atmospheric temperature for 20 min. The process was repeated thrice after transferring the roots to a new sterile centrifuge tube. The roots were removed, and the water on its surface was cleaned with sterile filter paper. Tissue samples were immediately placed in liquid nitrogen for full grinding. Total DNA from roots was extracted from 0.02 g freeze crushed tissue using the plant genomic DNA extraction kit (DP305; Tiangen, Beijing, China). Its purity was determined using the NanoDrop 2000 micro-ultraviolet spectrophotometer (Thermo Fisher Scientific, Waltham, MA, Unites States).

### 2.4. High-throughput sequencing of tomato root bacteria

The V5–V7 region of bacterial 16S ribosomal RNA (rRNA) gene was amplified through polymerase chain reaction (PCR) using universal primers: 799F (5′-AACMGGATTAGATACCCKG-3′) and 1193R (5′-ACGTCATCCCCACCTTCC-3′), and the barcode sequence, provided by Majorbio Biotechnology Co. Ltd. (Shanghai, China) was added to the 5′ end of the PCR product. The PCR reaction system was 50 μL, with 50 ng of total DNA from tomato root template, 1.5 μL of 10 μmol/L of 5′ and 3′ end primers respectively, 5 μL of 2 mmol/L dNTPs, 2 μL of MgSO_4_, 5 μL of 10× KOD buffer, 1 μL of KOD Plus, and added ddH_2_O to 20 μL. PCR reaction conditions were as follows: 95°C for 3 min; 30 cycles at 95°C for 30 s, 63°C for 30 s, 68°C for 30 s; and finally 68°C for 5 min. Each sample was repeatedly amplified by PCR thrice, and the three PCR products were mixed. The PCR products were cut and purified using an AxyPrepDNA gel recovery kit (Axygen Biosciences, Union City, CA, USA), and the DNA concentration was detected using 2% agarose electrophoresis. PCR products were detected and quantified using the QuantiFluor™-ST blue fluorescence quantitative system (Promega, Madison, WI, USA) and mixed according to the sequencing quantity of each sample. Majorbio Biotechnology Co. Ltd. (Shanghai, China) generated libraries for sequencing on Miseq PE300 (Illumina, San Diego, CA, United States).

### 2.5. Data analysis of high-throughput sequencing of tomato root bacteria

Paired-end (PE) reads obtained by Miseq sequencing were first spliced according to the overlapping relationship, and the sequence quality was controlled and filtered simultaneously. The Quantitative Insights into Microbial Ecology (QIIME) software package was used for quality assessment of sequencing data and subsequent data analysis (Caporaso et al., 2010). Sequences with less than 200 bp length, fuzzy bases, and mismatched primers of more than two bases were excluded. UPARSE (version 7.0.1090, http://drive5.com/uparse/) was used to extract the non-duplicate sequences from the optimized sequences, and the non-duplicate sequences were clustered through operational taxonomic units (OTU) based on 97% similarity (Edgar, 2010). The ribosomal database project (RDP) classifier (version 2.11, http://sourceforge.net/projects/rdp-classifier/) was used for taxonomic analysis with a confidence threshold of 0.7, and the SILVA database^1^ (Quast et al., 2013) was used for taxonomic annotation to obtain OTU datasets. Species (OTU) with sequence numbers >10 in at least six samples and total sequence numbers ≥60 were reserved and leveled according to the minimum sample sequence number (14,351 reads), and an OTU table was generated for subsequent analysis. The adequacy of sequencing samples was evaluated according to the rank-abundance curves, Pan/Core curves, and Rarefaction curves (based on Shannon index).

Sobs, Chao, Ace, Shannon, and Simpson indexes in alpha diversity (*α*-diversity) and coverage index, which reflect the community coverage index, were used to analyze the microbial diversity of each treatment. *α*-diversity was calculated based on the OTU table obtained above using “alpha_rarefaction.py” in QIIME. The Student’s *t*-test was used to compare the statistical differences in α-diversity among the treatment groups. Beta diversity (*β*-diversity) used principal coordinates analysis (PCoA) (distance algorithm Bray_curits, inter-group difference test analysis of similarities (ANOSIM)) and non-metric multidimensional scaling (NMDS) analysis (distance algorithm Bray_curits, inter-group difference test analysis of dissimilarities (ADONIS)) to analyze the difference in microbial community composition among treatments. The Student’s *t*-test (two-tailed; FDR estimation; Confidence interval: Student’s inverted, conf. Level = 0.95) was used to compare the differences in genus abundance between two diverse groups of samples, and one-way ANOVA (FDR estimation; Post-hoc analysis: Tukey–Kramer, conf. Level = 0.95) was used to compare the differences in genus abundance among three diverse groups of samples.

The phylogenetic investigation of communities by reconstruction of unobserved states (PICRUSt) software package was used to predict the function of sequencing data (Langille et al., 2013). First, the OTU abundance table was normalized using PICRUSt. Then, the cluster of orthologous groups of proteins (COG) family information and Kyoto Encyclopedia of Genes and Genomes (KEGG) ortholog (KO) information corresponding to each OTU were obtained by the corresponding Greengene ID of each OTU. The COG and KO abundance were then calculated. The functions of each COG from the EggNOG database^2^ were analyzed to obtain the functional abundance spectrum. The information on KO, pathway, and enzyme commission (EC) was obtained using the KEGG database.^3^ Basing on this, the abundance difference of metabolic pathways at the third level among different comparison groups was statistically compared with one-way ANOVA (Tukey–Kramer, conf. Level = 0.95), and the statistically significant differences were screened. Further, the functional categories of cellular process, environmental information processing, genetic information processing, metabolism, organismal systems, and others were counted. Then, the significant (≥ 2-fold) upregulation fold of metabolism pathways was calculated.

## 3. Results

### 3.1. Annotation and evaluation of the bacterial community in tomato root for each treatment

In total, 702,776 optimized sequences with an average length of 375 bp were obtained for 16S rRNA high-throughput sequencing of tomato root bacteria, reflecting the V5–V7 region of 16S rRNA (Supplementary Table S1). The OTU data were screened, and those with sequence numbers ≥10 in at least six samples and the sum of the sequence numbers ≥60 were reserved. The reserved OTUs were flattened according to the minimum sample sequence number to obtain an OTU table for subsequent analysis. The OTUs were identified in the taxonomic notes, belonging to 12 phyla, 14 classes, 41 orders, 58 families, and 109 genera.

**Figure 1:**
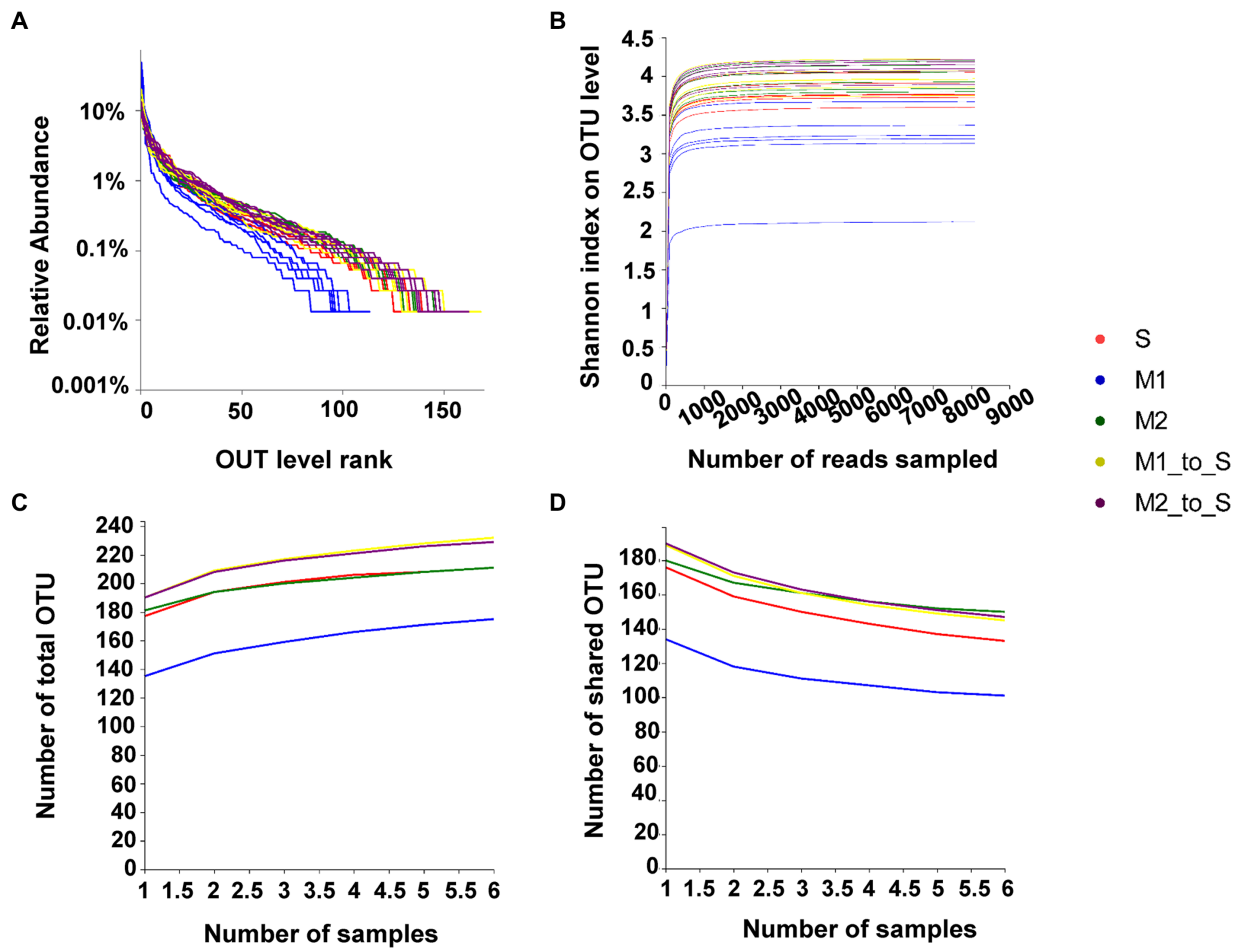
Evaluation of bacterial community in tomato root of each treatment. **(A)** Rank-Abundance curves, **(B)** Rarefaction curves (based on Shannon index), **(C)** Pan analysis, **(D)** Core analysis. S, soil cultivation; M1, substrate 1 cultivation; M2, substrate 2 cultivation; M1_to_S, substrate 1 transplanted to soil cultivation; M2_to_S, substrate 2 transplanted to soil cultivation.

The rank-abundance curve showed that each treatment curve gently decreased on the horizontal axis, indicating a relatively uniform distribution of each treatment species (Figure 1A). The Shannon curve for each treatment tended to be flat, indicating that the data volume met the sequencing requirements, and the microbial diversity information of each treatment was reflected more comprehensively (Figure 1B). The Core/Pan analysis demonstrated that the increase or decrease in the total OTU number (Figure 1C) and shared OTU number (Figure 1D) of each treatment declined and flattened, indicating that the sample size of each treatment met the requirements for species richness and core species number assessment.

### 3.2. *α*-Diversity differences in the bacterial community in tomato seedling root among different culture media

**Figure 2:**
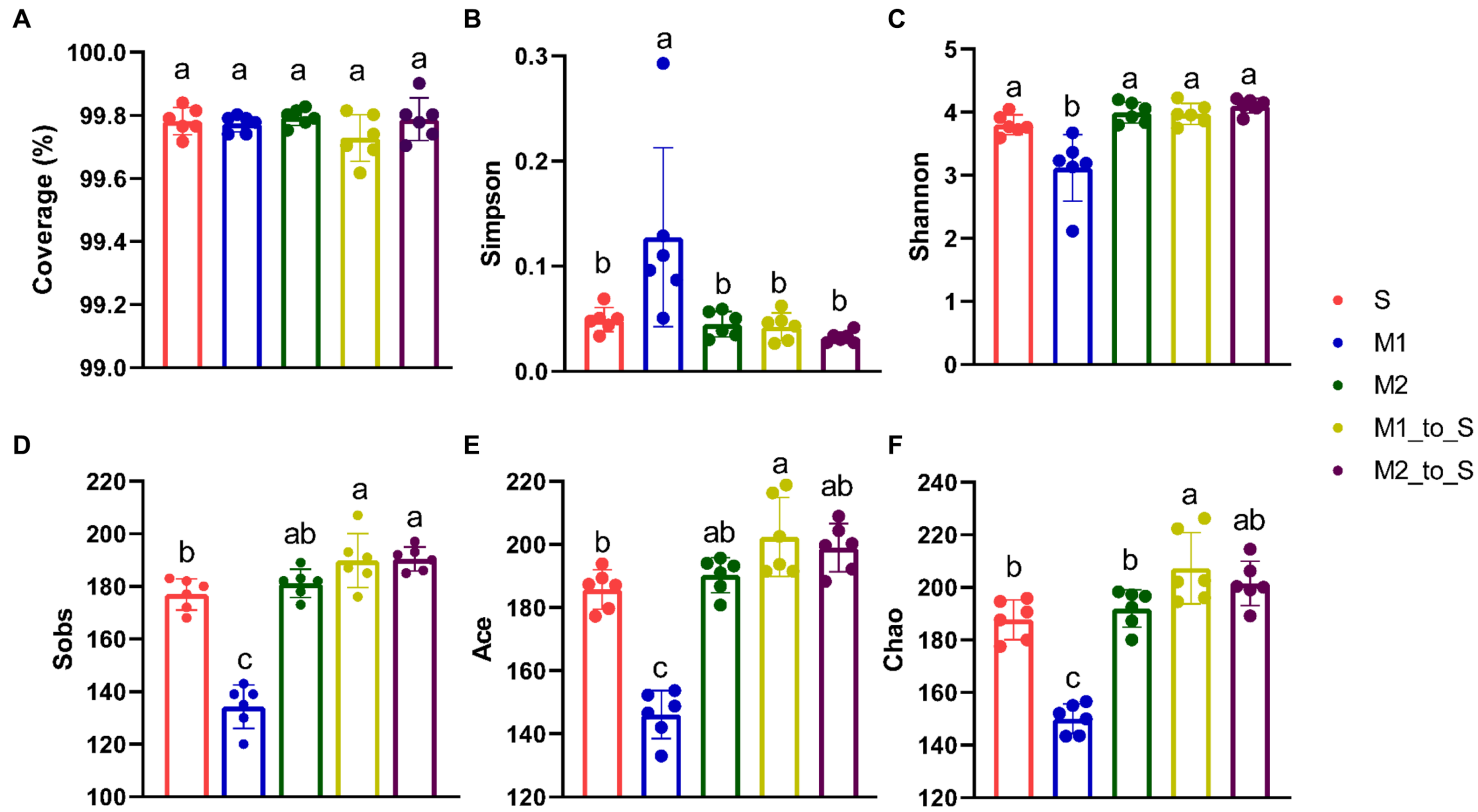
α-diversity differences on OTU level in the bacterial community in tomato seedling root among different culture media. **(A)** Coverage index, **(B)** Simpson index, **(C)** Shannon index, **(D)** Sobs index, **(E)** Ace index, **(F)** Chao index. The error bar represents the standard deviation (*n* = 3), different lowercase letters on the column indicate significant difference between groups (*p* < 0.05, Duncan’s method). S, soil cultivation; M1, substrate 1 cultivation; M2, substrate 2 cultivation; M1_to_S, substrate 1 transplanted to soil cultivation; M2_to_S, substrate 2 transplanted to soil cultivation.

The *α*-diversity indicators of the bacterial community in each treatment were analyzed. All coverage indices were greater than 99%, indicating that this sequencing data was sufficient to guarantee the reliability of subsequent diversity analysis (Figure 2A). The community diversity in the three cultivation media was different (Figures 2B–F). The differences in Simpson, Shannon, Sobs, Ace, and Chao indexes of microbial community between Group S and Group M2 were not statistically significant (*p <* 0.05), but the difference was larger than that in Group M1. Among them, the Simpson index in the M1 group was significantly higher than those in S and M2 groups (*p <* 0.05), whereas Shannon, Sobs, Ace, and Chao indexes in M1 were significantly lower than those in S and M2 groups (*p <* 0.05). The abundance of root bacteria in M1 was significantly lower than that in S and M2 (*p <* 0.05), and the evenness of the former was significantly higher than that in the latter (*p <* 0.05). The Sobs, Ace, Chao, and Shannon indexes in the M1_to_S group were significantly higher than those in the M1 group (*p <* 0.05). In contrast, the Simpson index was significantly decreased (*p <* 0.05), suggesting that the abundance of microflora increased while the uniformity decreased after transplanting from substrate 1 to the soil. The *α*-diversity indexes of the M2_to_S group showed no significant changes (*p <* 0.05), indicating no significant effect on community diversity after transplantation from substrate 2 to the soil. The Sobs, Ace, and Chao indexes, which characterize abundance, were all significantly increased in the M1_to_S group compared with the microflora that continuously grew in the soil (*p <* 0.05). In the M1_to_S group, the Sobs index, which was used to characterize richness, was significantly increased (*p <* 0.05), whereas the Chao index was slightly increased. There was no significant change in the Simpson index, which was used to characterize uniformity after the two substrates were transplanted into the soil. Overall, the *α*-diversity analysis indicated that both substrates increased community abundance but negatively or insignificantly affected community uniformity.

### 3.3. *β*-Diversity differences in the bacterial community in tomato seedling root among different culture media

**Figure 3:**
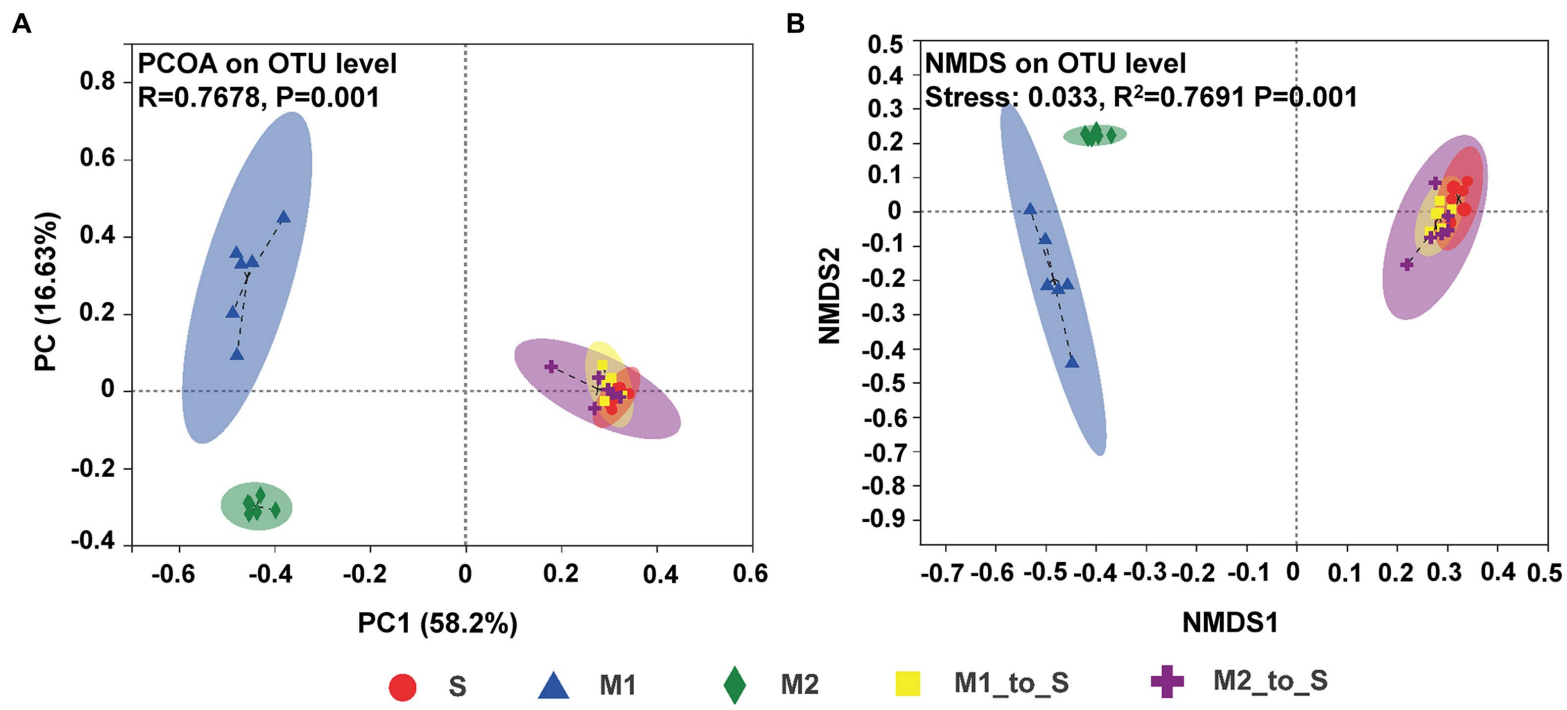
*β*-diversity differences on OTU level in the bacterial community in tomato seedling root among different culture media, **(A)** PcoA plot (ANOSIM for inter-group difference test, displacement number = 999, *R* = 0.7678, *p* = 0.001), **(B)** NMDS plot (ADONIS for inter-group difference test, displacement number = 999, *R*^2^ = 0.7691, and *p* = 0.001). S, soil cultivation; M1, substrate 1 cultivation; M2, substrate 2 cultivation; M1_to_S, substrate 1 transplanted to soil cultivation; M2_to_S, substrate 2 transplanted to soil cultivation.

The similarities or differences in the composition of bacterial communities in tomato seedling roots in different cultivation media were studied through the PCoA and NMDS analysis. PCoA (Figure 3A) showed that the microbiota in S, M1, and M2 groups were each clustered and within different confidence elliptical groups, with statistical differences (ANOSIM for inter-group difference test, displacement number = 999, *R* = 0.7678, *p* = 0.001). After being transferred from the two substrates to the soil, the root bacterial composition and abundance in the M1_to_S and M2_to_S groups were significantly different from the microflora in their respective primary substrate environments and had higher similarity to the soil microflora, which, however, was not statistically different. The results of the NMDS analysis (Figure 3B) were similar to those of PCoA; the microbiota in S, M1, and M2 groups were each clustered and showed statistical differences (ADONIS for inter-group difference test, displacement number = 999, *R*^2^ = 0.7691, and *p* = 0.001). After the two substrates were transferred to the soil, the similarity in bacterial community characteristics and soil microflora was higher in the M1_to_S group and the M2_to_S group.

### 3.4. Species composition and abundance differences in the bacterial community in tomato seedling root at genus level among different cultivation media

**Figure 4:**
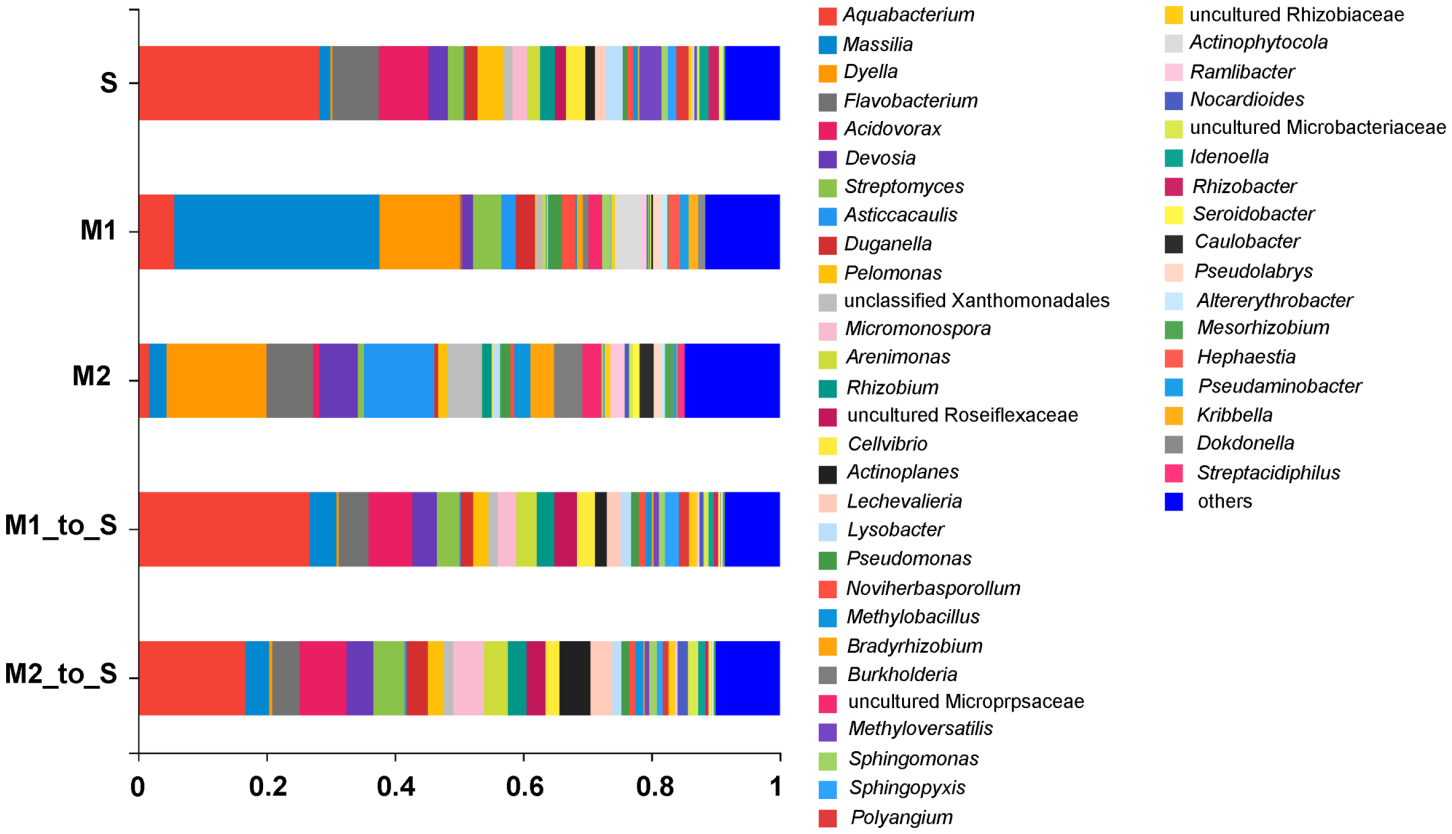
Species composition of bacterial community in tomato seedling root on genus level. S, soil cultivation; M1, substrate 1 cultivation; M2, substrate 2 cultivation; M1_to_S, substrate 1 transplanted to soil cultivation; M2_to_S, substrate 2 transplanted to soil cultivation.

The strain composition in each culture medium was comparatively analyzed at the genus level. The OTUs, belonging to the same genus from each group, were combined, and the species with an abundance ratio of <0.01% in all samples were classified as “others,” and finally obtain a column diagram of the bacterial community. The bacterial community diversities in S, M1, and M2 groups were insignificantly different at the genus level. However, the abundance differences among different genera were obvious (Figure 4). After the two substrates were transplanted into the soil, the bacterial structures and abundance in M1_to_S and M2_to_S groups were more similar to the soil environment at the genus level. In particular, the genera identified and obtained from each cultivation medium mainly include *Aquabacterium*, *Massilia*, *Dyella*, *Flavobacterium*, *Acidovorax*, *Devosia*, *Streptomyces*, *Asticcacaulis*, *Duganella*, *Pelomonas*, *Micromonospora*, *Arenimonas*, *Rhizobium*, *Cellvibrio*, *Actinoplanes*, *Lechevalieria*, *Lysobacter*, *Pseudomonas*, *Noviherbaspirillum*, *Methylobacillus*, *Bradyrhizobium*, *Burkholderia*, *Methyloversatilis*, *Sphingomonas*, *Sphingopyxis*, *Polyangium*, *Actinophytocola*, *Ramlibacter*, *Nocardioides*, *Idenoella*, *Rhizobacter*, *Seroidobacter*, *Caulobacter*, *Pseudolabrys*, *Altererythrobacter*, *Mesorhizobium*, *Hephaestia*, *Pseudaminobacter*, *Kribbella*, *Dokdonella*, *Streptacidiphilus*, and some genera in the family Xanthomonadales, Roseiflexaceae, Micropepsacese, Rhizobiaceae, and Microbacteriaceae.

**Figure 5:**
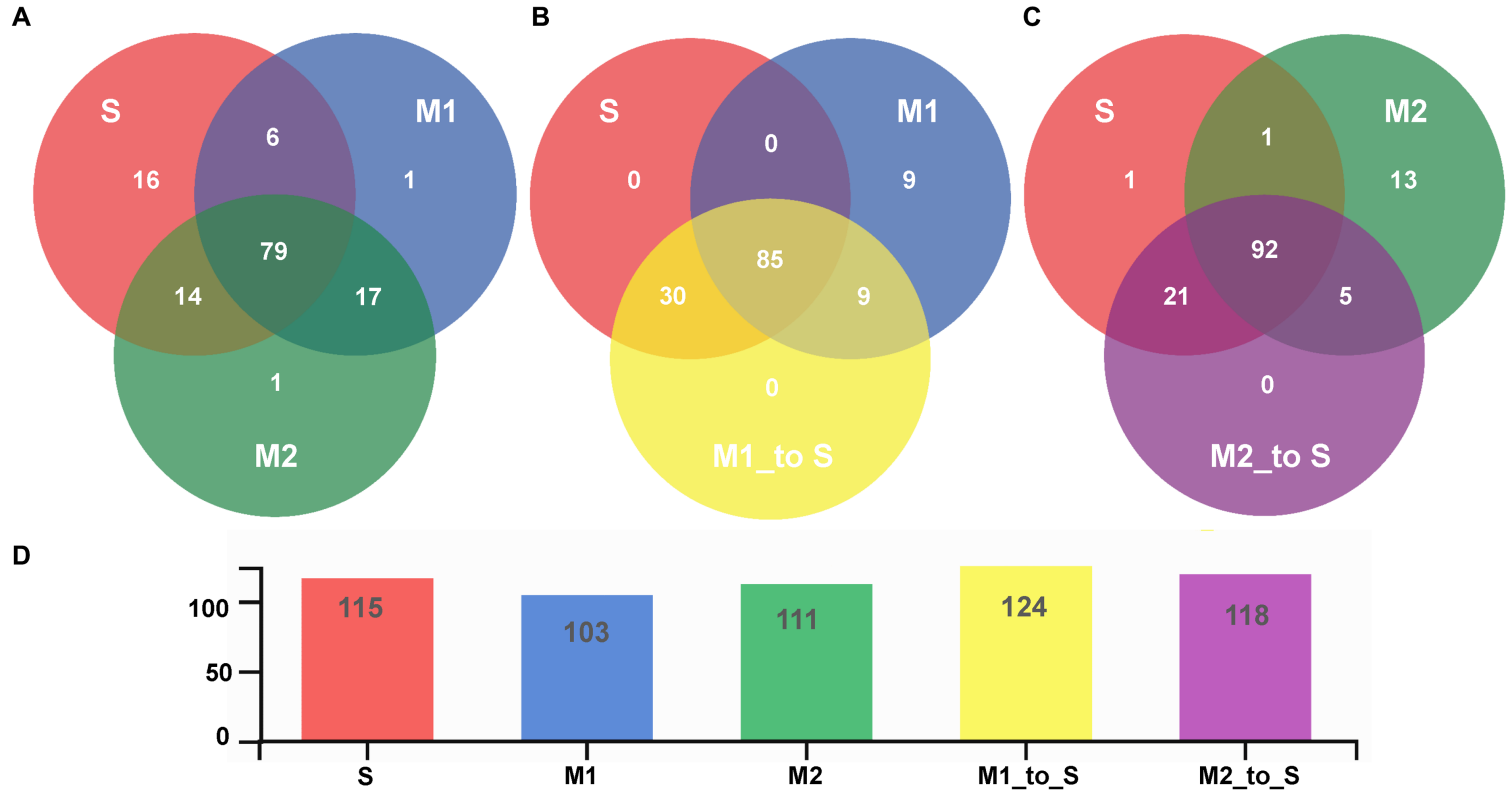
Venn diagram for root bacterial community composition in tomato seedling root on genus level **(A)** Comparison among S, M1 and M2 group, **(B)** Comparison among S, M1 and M1_to_S group, **(C)** Comparison among S, M2 and M2_to_S group, **(D)** Bar graph of the total number of genera of each treatment group. S, soil cultivation; M1, substrate 1 cultivation; M2, substrate 2 cultivation; M1_to_S, substrate 1 transplanted to soil cultivation; M2_to_S, substrate 2 transplanted to soil cultivation.

Furthermore, the common or unique genera of the bacterial community in different cultivation media were analyzed through a Venn diagram (Figure 5). First, the bacterial community of the S group was compared with that of the M1 and M2 groups. There were 79 bacterial genera in the tomato root with two substrates in the soil environment, accounting for 68.70% of the total genera in the soil, 76.70% of substrate 1, and 71.17% of substrate 2 (Figure 5A, Supplementary Table S2). Common genera were *Massilia*, *Roseomonas*, *Devosia*, *Pseudonocardia*, *Bacillus*, *Sphingomonas*, *Nocardioides*, *Rhodoplanes*, *Pseudolabrys*, *Pseudaminobacter*, *Dongia*, *Actinophytocola*, *Marmoricola*, *Sphingopyxis*, *Hyphomicrobium*, *Dyella*, *Ramlibacter*, *Caulobacter*, *Asticcacaulis*, *Pseudorhodoplanes*, *Reyranella*, *Ralstonia*, *Amycolatopsis*, *Rhizobium*, *Arenimonas*, *Mesorhizobium*, *Bradyrhizobium*, *Noviherbaspirillum*, *Methylobacillus*, *Steroidobacter*, *Limnobacter*, *Methyloversatilis*, *Phenylobacterium*, *Novosphingobium*, *Ensifer*, *Acidovorax*, *Streptomyces*, *Pseudomonas*, *Bosea*, *Brevundimonas*, *Peredibacter*, *Kribbella*, *Pelomonas*, *Flavobacterium*, *Ideonella*, *Altererythrobacter*, *Ferrovibrio*, *Acidibacter*, *Pseudarthrobacter*, *Rubrivivax*, *Aeromicrobium*, *Mycobacterium*, *Bordetella*, and an unidentified bacteria. In addition, there were 16 unique genera in S, one in M1, and one in M2. Groups S and M1 were compared with group M1_to_S (Figure 5B, Supplementary Table S3). The results showed that all the genera in the S group existed in the M1 group after transplantation. In contrast, nine genera in the M1 group (including *Chujaibacter*, *Streptacidiphilus*, *Bauldia*, *Bryobacter*, *Acidothermus*, *Tistrella*, *Granulicella*, and two unidentified genera) did not exist in group M1_to_S. All the genera in group M1_to_S were derived from group S or group M1. The comparison between groups S, M2, and M2_to_S revealed that, after transplantation, all the genera in the S group, except for one genus from class Bacteroidia, were present in M2_to_S (Figure 5C, Supplementary Table S4). In contrast, 13 genera in M2 (including *Chujaibacter*, *Streptacidiphilus*, *Gemmatimonas*, *Dokdonella*, *Soilmonas*, *Bauldia*, *Hephaestia*, *Bryobacter*, *Paludibaculum*, *Tistrella*, *Granulicella*, and *Acidisphaera*) did not exist in the M2_to_S group, and all genera in the M2_to_S group were derived from the S group or the M2 group.

**Figure 6:**
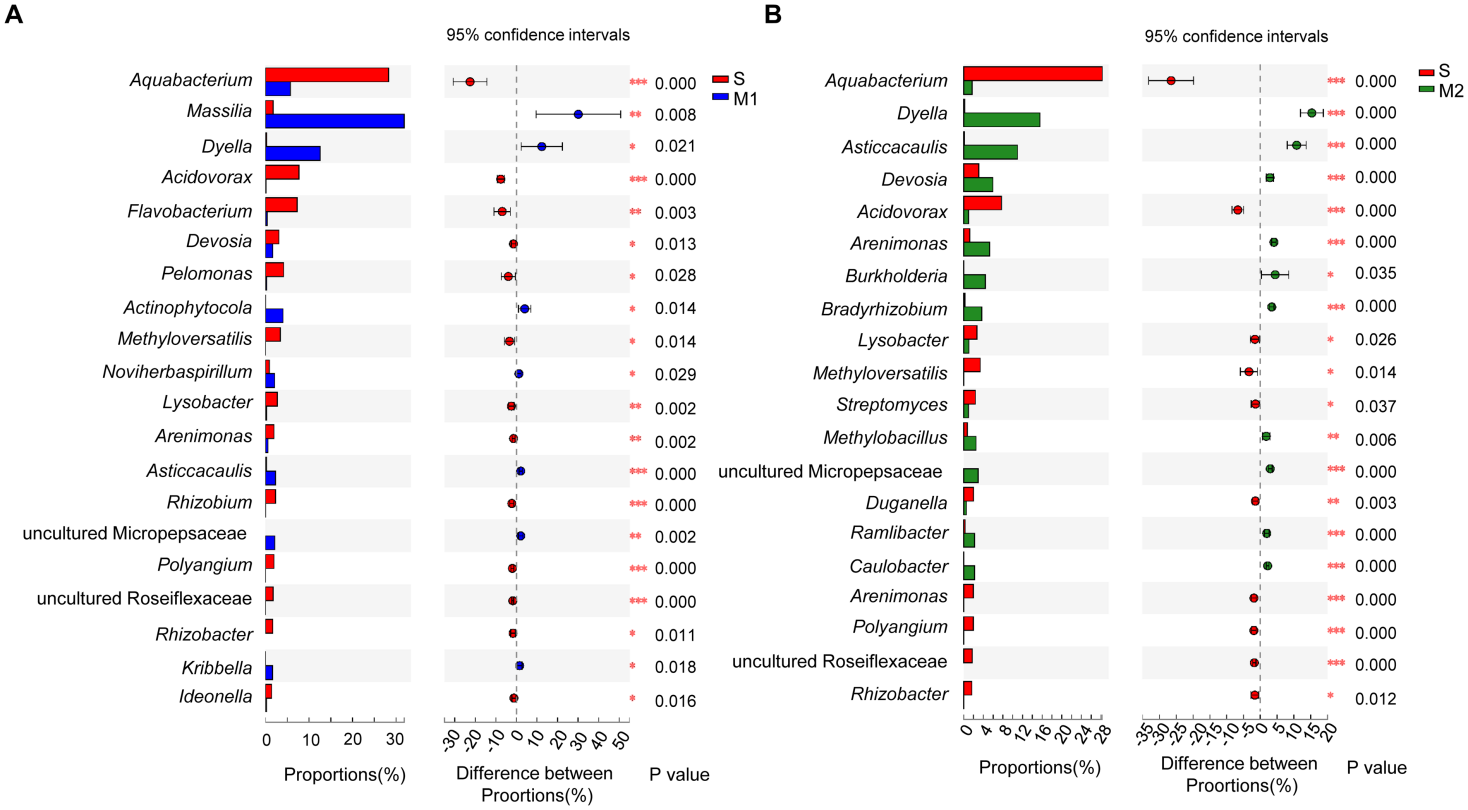
Comparison of genus abundance (Top 20) in root bacterial community in tomato seedling root between different cultivation media. **(A)** S group vs. M1 group, **(B)** S group vs. M2 group. The Student’s *t*-test (two-tailed; FDR estimation; Confidence interval: Student’s inverted, conf. Level = 0.95) was used in the comparison. S, soil cultivation; M1, substrate 1 cultivation; M2, substrate 2 cultivation. “*” at the right of each group of bars in (A, B) present significant difference (*p < 0.05; **p < 0.01).

**Figure 7:**
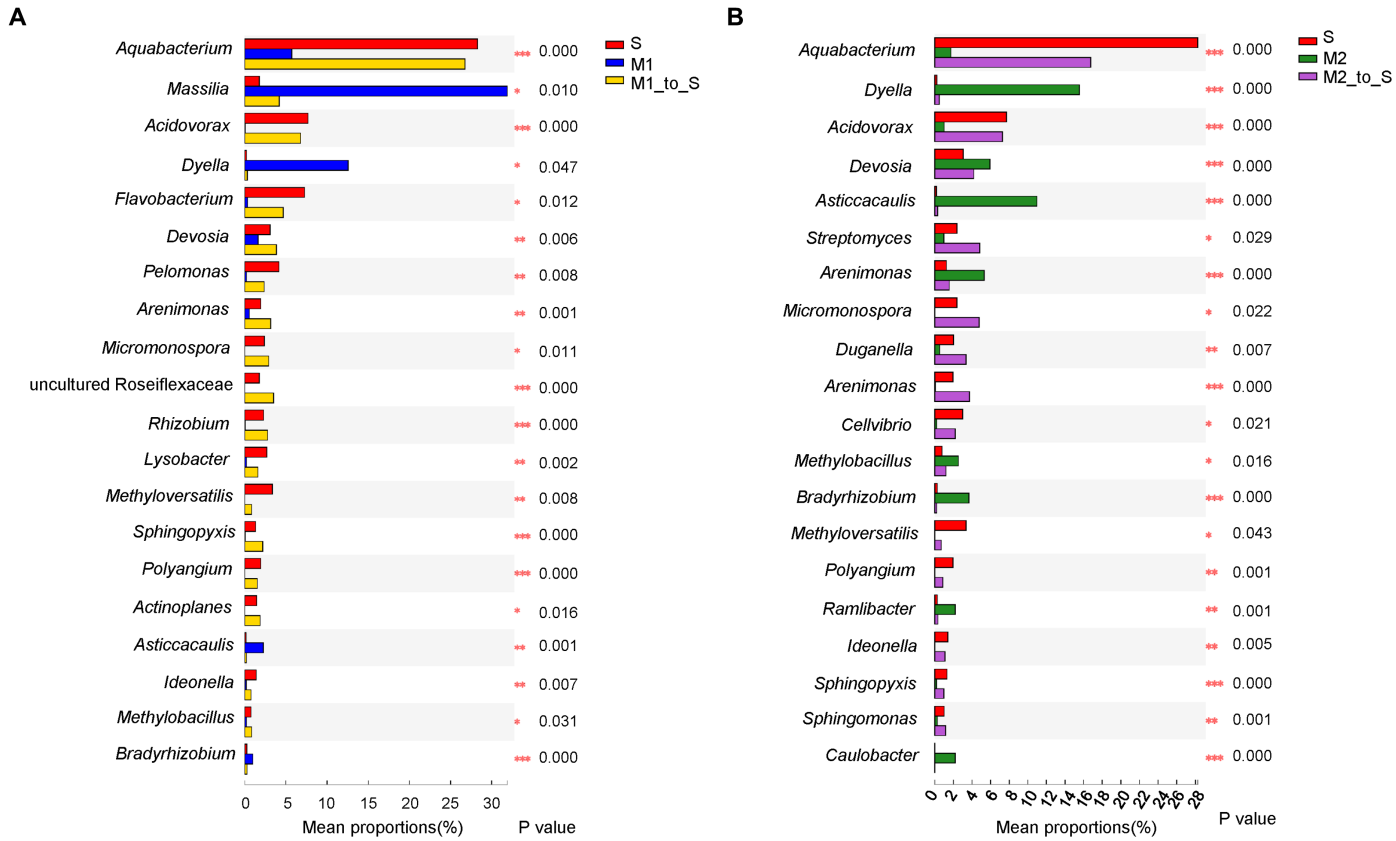
Comparison of genus abundance in root bacterial community in tomato seedling root between different cultivation media. **(A)** Comparison among S, M1 and M1_to_S group, **(B)** Comparison among S, M2 and M2_to_S group. One-way ANOVA (FDR estimation; Post-hoc analysis: Tukey–Kramer, conf. Level = 0.95) was used in the comparisons. S, soil cultivation; M1, substrate 1 cultivation; M2, substrate 2 cultivation; M1_to_S, substrate 1 transplanted to soil cultivation; M2_to_S, substrate 2 transplanted to soil cultivation. “*” at the right of each group of bars in (A, B) present significant difference (*p < 0.05; **p < 0.01).

In addition, the significant difference in the community abundance data (the top 20 genera in abundance) under different cultivation media was assessed through the significance test of inter-group difference (*p <* 0.05). First, the abundance of group S was compared with that of the M1 and M2 groups each (Figure 6A). Compared with group M1, the abundance of group S was significantly higher than that of the group M1 in genera *Aquabacterium*, *Acidovorax*, *Flavobacterium*, *Devosia*, *Pelomonas*, *Methyloversatilis*, *Lysobacter*, *Arenimonas*, *Rhizobium*, *Polyangium*, *Rhizobacter*, *Ideonella*, and a genus from family Roseiflexaceae. The genera in which the abundance of group S was significantly lower than that of group M1 were *Aquabacterium*, *Acidovorax*, *Flavobacterium*, *Devosia*, *Pelomonas*, *Methyloversatilis*, *Lysobacter*, *Arenimonas*, *Rhizobium*, *Polyangium*, *Rhizobacter*, *Ideonella*, and a genus of Micropepsaceae. The abundance of the S group was significantly higher than that of the group M2, belonging to the genera *Aquabacterium*, *Acidovorax*, *Lysobacter*, *Methyloverriding*, *Streptomyces*, *Duganella*, *Arenimonas*, *Polyangium*, *Rhizobacter*, and a genus of Roseiflexaceae (Figure 6B). The abundance of the group S was significantly lower than that of the group M2 in *Dyella*, *Asticcacaulis*, *Devosia*, *Arenimonas*, *Burkholderia*, *Bradyrhizobium*, *Methylobacillus*, *Ramlibacter*, *Caulobacter*, and a genus from family Roseiflexaceae. Besides, the abundance differences between the corresponding substrate and soil after tomato transplantation were analyzed. Among the top 20 abundant genera, the abundance of the microflora in most of the M1_to_S groups was significantly increased compared with that in the M1 group (Figure 7A), which included *Aquabacterium*, *Acidovorax*, *Flavobacterium*, *Devosia*, *Pelomonas*, *Arenimonas*, *Micromonospora*, *Rhizobium*, *Lysobacter*, *Methyloversatilis*, *Sphingopyxis*, *Actinoplanes*, *Ideonella*, *Methylobacillus*, and a genus from Roseiflexaceae. The abundance of the microflora in a small fraction of the M1_to_S group was significantly reduced compared to that in the M1 group, including *Massilia*, *Dyella*, *Asticcacaulis*, and *Bradyrhizobium*. Among the top 20 abundant genera, the abundance of microflora in most of the M2_to_S group was also significantly increased compared with that in the M2 group (Figure 7B), including *Aquabacterium*, *Acidovorax*, *Streptomyces*, *Micromonospora*, *Duganella*, *Arenimona*, *Cellvibrio*, *Methyloversatilis*, *Polyangium*, *Ideonella*, *Sphingopyxis*, and *Sphingomonas*. In addition, the abundance of microflora in the M2_to_S group was significantly reduced than that in the M2 group, including *Dyella*, *Devosia*, *Asticcacaulis*, *Arenimonas*, *Methylobacillus*, *Bradyrhizobium*, *Ramlibacter*, and *Caulobacter*. The abundance of soil microflora after transplantation was similar to that of soil microflora transplanted from substrate 1 and substrate 2. The results suggest that if the abundance of a genus in the soil was higher than that in the substrate, the abundance of the genus after transplantation would also be significantly higher than that in the substrate. In contrast, if the abundance of a genus in the soil was lower than that in the substrate, the abundance of the genus after transplantation would also be significantly lower than that in the substrate.

### 3.5. Differences in metabolic pathways of bacterial communities in tomato seedling root between different cultivation substrates

**Figure 8:**
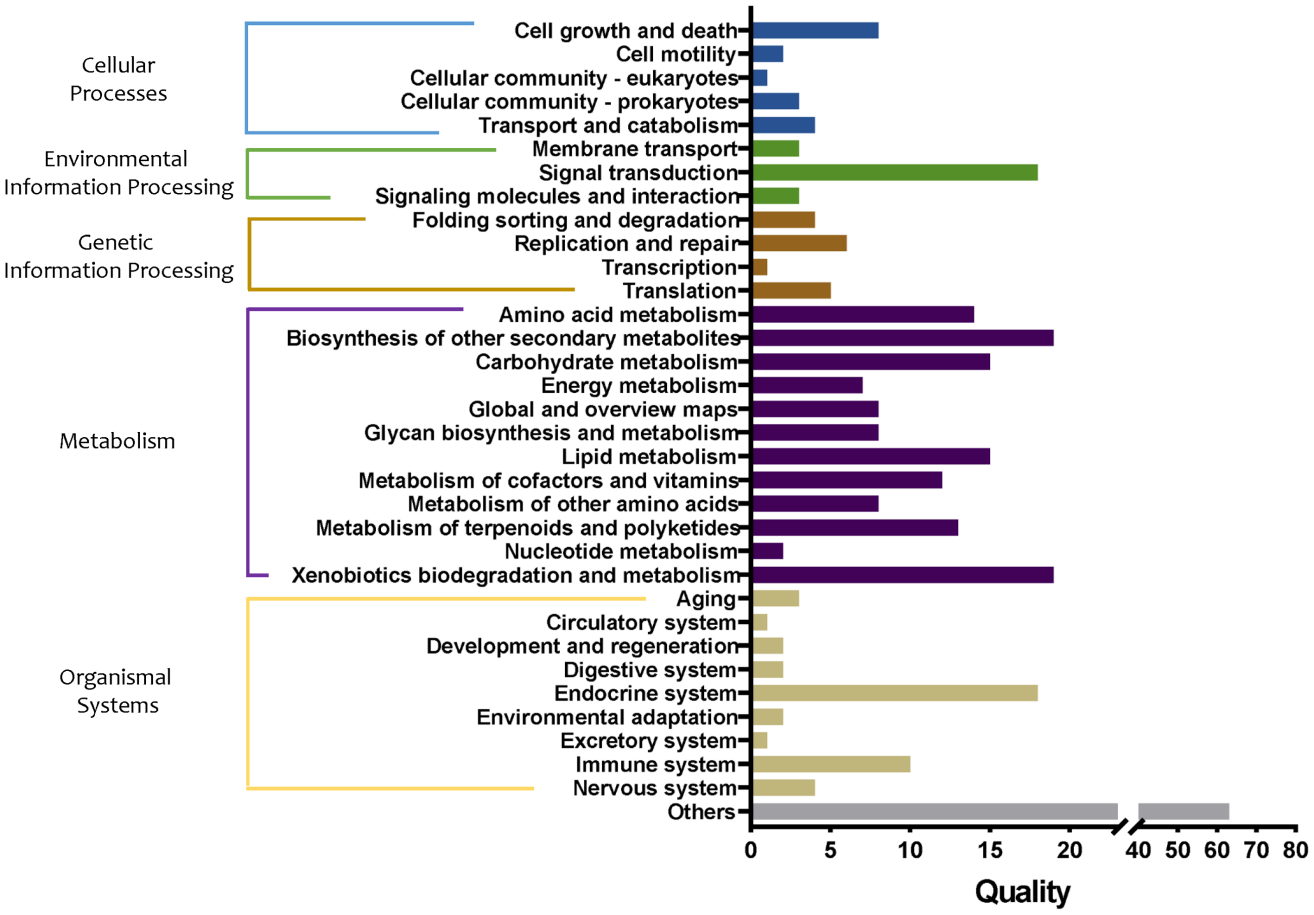
Quality of metabolic pathways in root bacterial community, which were significantly different among S, M1, and M2 groups (conf. Level = 0.95). S, soil cultivation; M1, substrate 1 cultivation; M2, substrate 2 cultivation.

The abundance of metabolic pathways in bacteria in tomato seedling roots cultured in different grafting media was compared, and the main functions of the bacteria were predicted and analyzed. First, the differences in metabolic pathways of bacteria in soil and two substrates were compared (Figure 8). The proxy pathways with significant differences among the three cultivation media mainly included 18 in cellular processes, 24 in environmental information processing, 16 in genetic information processing, 140 in metabolism, 43 in organizational systems, and 63 in other pathways. Among them, the differences in the metabolic pathways of metabolism were the largest, mainly involving the metabolism of amino acids, carbohydrates, energy, lipid cofactors, vitamins, other amino acids, terpenoids and polyketides, and nucleotide, and the synthesis and metabolism of glycan and xenobiotic and other secondary metabolites. In particular, compared with group S, the abundance of synthetic pathways of sesquiterpenoid, triterpenoid, and arabinogalactan in group M1 increased by 2.33 and 2.30 times, respectively, whereas that of the degradation pathway of bisphenol and furfural increased by 2.09 and 2.01 times, respectively. In addition, the abundance of pathways of biosynthesis of 12-, 14-, and 16-membered macrolides (43.49-fold decrease), biosynthesis of enediyne antibiotics (10.04-fold decrease), biosynthesis of various secondary metabolites (9.37-fold decrease), fatty acid elongation (6.28-fold decrease), flavonoid biosynthesis (5.79-fold decrease), stilbenoid, diarylheptanoid, and gingerol biosynthesis (5.79-fold decrease), type I polyketide structures (3.51-fold decrease), and nitrotoluene degradation (2.18-fold decrease) pathway abundance in the group M1 were markedly decreased, respectively. Compared with the S group, monoterpenoid biosynthesis (33.33-fold increase), furfural degradation (11.12-fold increase), bisphenol degradation (4.57-fold increase), sesquiterpenoid, and triterpenoid biosynthesis (2.84-fold increase), other glycan degradation (2.32-fold increase), staurosporine biosynthesis (2.27-fold increase), steroid hormone biosynthesis (2.18-fold increase), N-glycan biosynthesis (2.17-fold increase), glycosphingolipid biosynthesis (2.11-fold increase), sphingolipid metabolism (2.10-fold increase), and various types of N-glycan biosynthesis (2.10-fold increase) pathway abundance in the group M2 was markedly increased, respectively. Furthermore, biosynthesis of enediyne antibiotics (27.19-fold decrease), biosynthesis of 12-, 14-, and 16-membered macrolides (24.95-fold decrease), fatty acid elongation (17.28-fold decrease), flavonoid biosynthesis (7.08-fold decrease), stilbenoid, diarylheptanoid, and gingerol biosynthesis (7.08-fold decrease), type I polyketide structures (5.27-fold decrease), and biosynthesis of various secondary metabolites pathways (3.00-fold decrease) pathway abundance in group M2 were markedly decreased, respectively.

**Figure 9:**
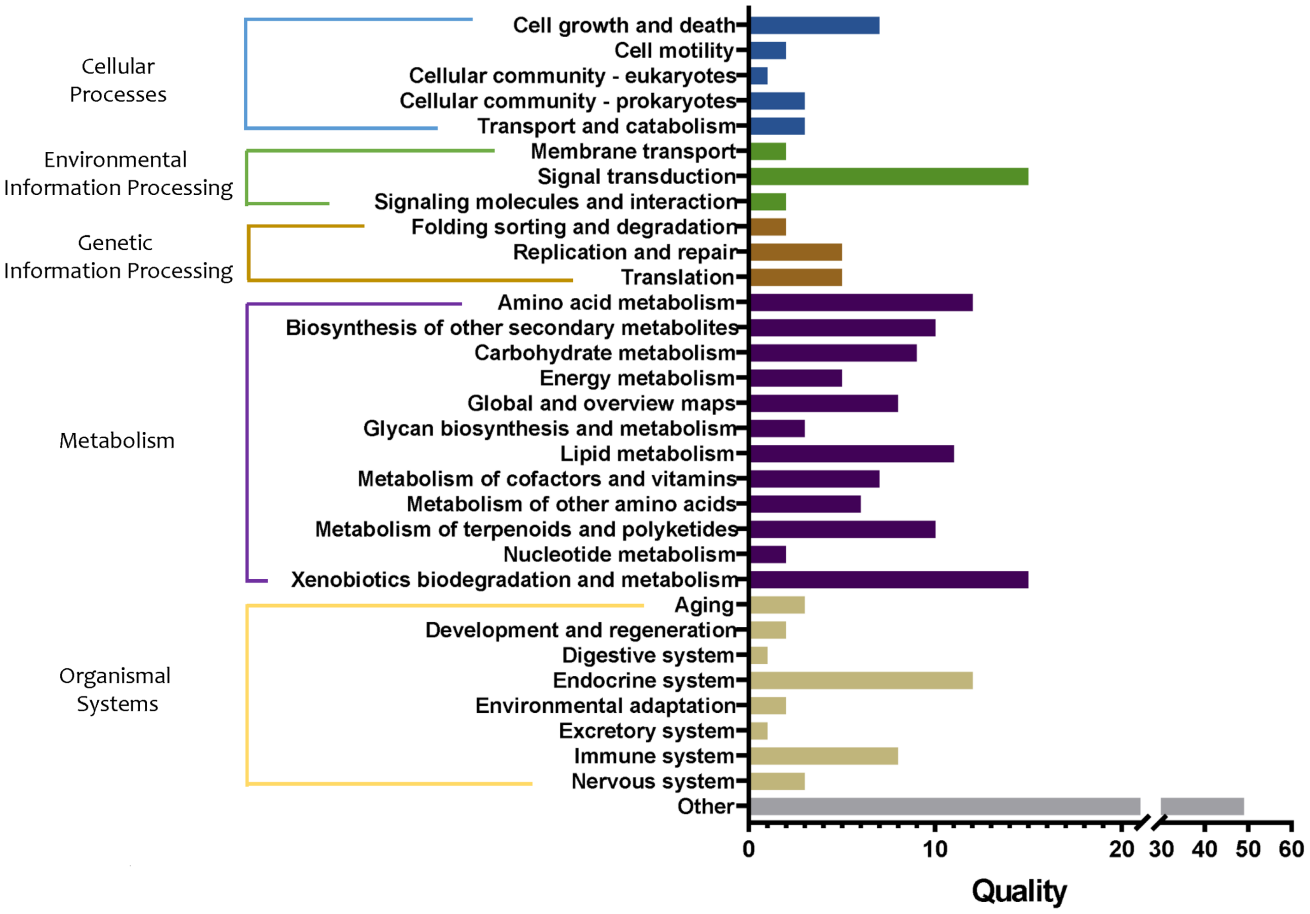
Quality of metabolic pathways in root bacterial community, which were significantly different among S, M1, and M1_to_S groups (conf. Level = 0.95). S, soil cultivation; M1, substrate 1 cultivation; M1_to_S, substrate 1 transplanted to soil cultivation.

Then, the influence of bacterial metabolic pathways in tomato roots after transplantation from the substrate to the soil was analyzed, and the groups S, M1, and M1_to_S were compared. The metabolic pathways in the three groups were significantly different, including 16 in cellular processes, 19 in environmental information processing, 12 in genetic information processing, 98 in metabolism, 32 in organismal systems, and 49 in other pathways (Figure 9). Among them, the differences in the metabolic pathways of metabolism were the largest, mainly involving the metabolism of amino acids, carbohydrates, energy, lipid cofactors, vitamins, other amino acids, terpenoids and polyketides, and nucleotide, and the synthesis and metabolism of glycan and xenobiotic, and the synthesis of other secondary metabolites. Specifically, compared with the M1 group, the biosynthesis of 12-, 14-, and 16-membered macrolides (59.91-fold increase), biosynthesis of enediyne antibiotics (12.98-fold increase), biosynthesis of various secondary metabolites (11.69-fold increase), flavonoid biosynthesis, the pathway abundance of stilbenoid (6.55-fold increase), diarylheptanoid and gingerol biosynthesis (6.55-fold increase), type I polyketide structures (4.15-fold increase), fatty acid elongation (3.84-fold increase), monoterpenoid biosynthesis (3.40-fold increase), and nitrotoluene degradation (2.02-fold increase) pathway abundance in the group M1_to_S were markedly increased, respectively. In addition, the abundance of the bisphenol degradation pathway in the group M1_to_S decreased by 2.68 times. Compared with group S, the abundance of monoterpenoid biosynthesis pathways in group M1_to_S increased by 3.37 times, and that of non-metabolism-related pathways decreased by more than 2 times.

**Figure 10:**
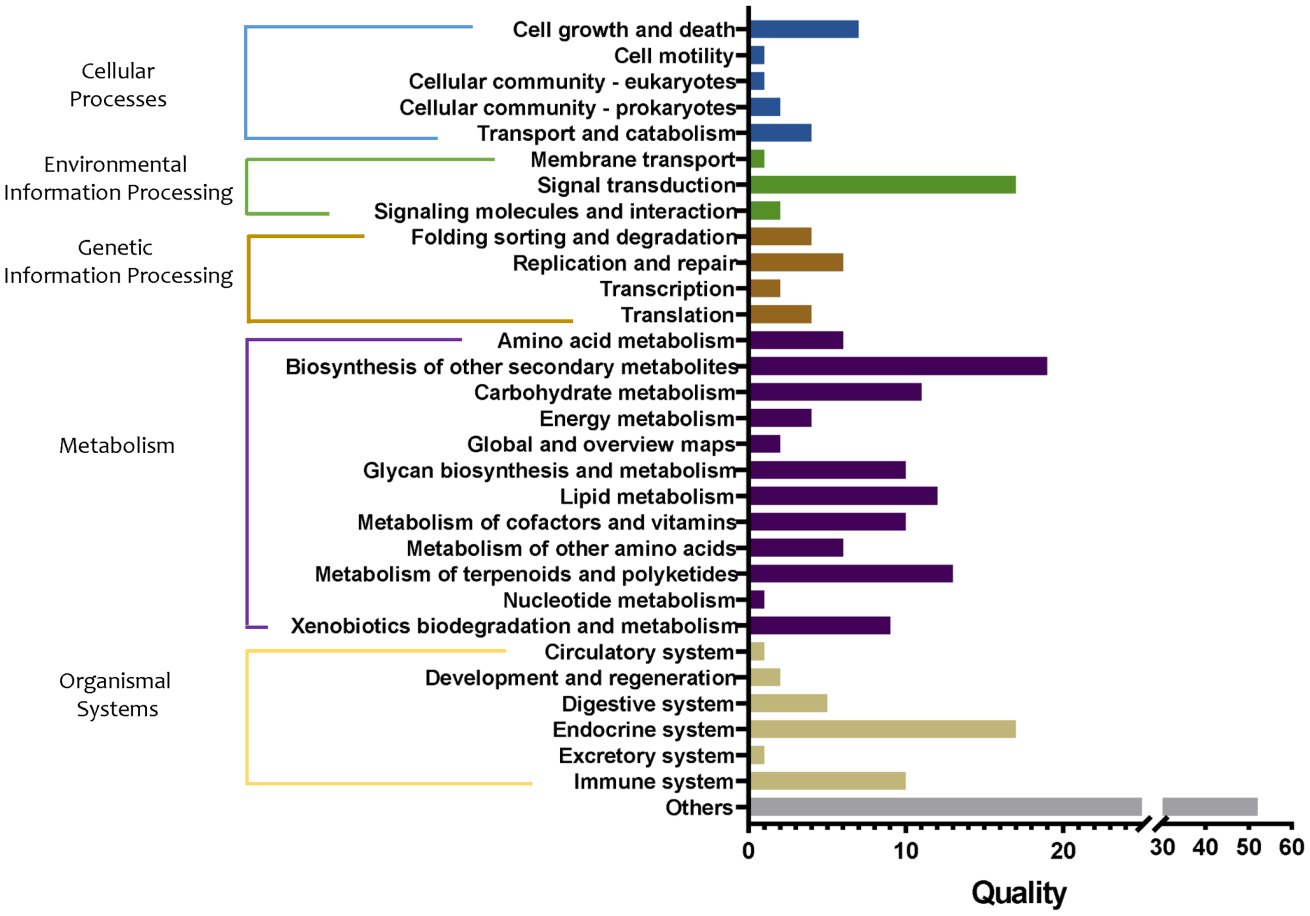
Quality of metabolic pathways in root bacterial community, which were significantly different among S, M2, and M2_to_S groups (conf. Level = 0.95). S, soil cultivation; M2, substrate 2 cultivation; M2_to_S, substrate 2 transplanted to soil cultivation.

When the S, M2, and M2_to_S groups were compared (Figure 10), 15 in cellular processes, 20 in environmental information processing, 16 in genetic information processing, 103 in metabolism, 36 in organismal systems, and 52 other pathways showed marked differences among the three groups. Among them, the differences in the metabolic pathways were the largest, mainly involving the metabolism of amino acids, carbohydrates, energy, lipid cofactors, vitamins, other amino acids, terpenoids and polyketides, nucleotide, and the synthesis and metabolism of glycan and xenobiotic, and the synthesis of other secondary metabolism. Specifically, compared with the M2 group, biosynthesis of enediyne antibiotics (66.52-fold increase), biosynthesis of 12-, 14-, and 16-membered macrolides (58.26-fold increase), flavonoid biosynthesis (15.01-fold increase), stilbenoid, diarylheptanoid, and gingerol biosynthesis (15.01-fold increase), fatty acid elongation (11.44-fold increase), type I polyketide structures (9.08-fold increase), biosynthesis of various secondary metabolites (5.58-fold increase), tetracycline biosynthesis (3.47-fold increase), caffeine metabolism (3.22-fold increase), biosynthesis of various secondary metabolites (3.15-fold increase), biosynthesis of type II polyketide backbone (2.99-fold increase), steroid degradation (2.97-fold increase), and lipoarabinomannan (lam) biosynthesis (2.79-fold increase) pathway abundance in the M2_to_S group were markedly increased, respectively. In addition, the abundance of furfural degradation, bisphenol degradation, and staurosporine biosynthesis pathways in the M2_to_S group decreased by 9.98, 4.62, and 2.42 times, respectively. Compared with the S group, the abundance of the monoterpenoid biosynthesis pathway in the M2_to_S group increased by 20.49 times, and the abundance of the non-metabolism-related pathway decreased by more than 2 times.

## 4. Discussion

The difference in tomato root bacteria community in different planting media and the effect of substrate transplantation to the soil on root bacteria community were investigated by amplicon-based high-throughput sequencing method. The *α*-diversity of substrate 1 community was significantly different from that of soil community, whereas that of substrate 2 community did not exhibit significant differences. However, *β*-diversity revealed that the differences in the number and abundance of the community of the three cultivation media were significant, and they were clustered into groups. According to Venn analysis, the same 79 genera accounted for 68.70, 76.70, and 71.17% of the total genera were existing in soil, substrate 1, and substrate 2 community, respectively. The above results were similar to the characteristic of the cucumber root bacteria community (Zhou et al., 2022). The results indicated that a batch of bacteria colonized on the tomato roots, which were relatively stable in different cultivation media; however, the abundance difference was significant. According to previous studies, plant root bacterial community composition originates from the horizontal transfer of the community in growth media (soil, substrate, etc.), and the vertical transfer of the flora carried by seeds (Shade et al., 2017). Currently, it is generally believed that under the influence of root exudates, after the seed roots come into contact with the soil, the soil microflora around the roots gradually differentiate into rhizosphere microbial community and gradually colonize and enrich in various regions of the roots (including the endosphere in the root layer or rhizosphere). Which was significantly affected by the morphology of roots and the quantity and quality of root exudates produced by different genotypes (Sasse et al., 2018). In addition, they are also affected by the plant’s immune system, developmental stage, and season (Hassani et al., 2018). In our study, the plant genotypes were the same, but most of the bacterial species (at genus level) in the roots were not altered when the cultivation medium was changed. Among the 79 genera shared by soil and two substrate bacterial community, *Devosia* (Chhetri et al., 2022), *Bacillus* (Salwan et al., 2021; Kannan et al., 2022), *Rhizobium* (Igiehon and Babalola, 2021), *Mesorhizobium* (Knežević et al., 2022), *Bradyrhizobium* (Sugiyama et al., 2015), *Ensifer* (Dang et al., 2019), *Pseudomonas* (Babalola et al., 2021), *Bosea* (Safronova et al., 2015), and *Brevundimonas* (Singh et al., 2016) are plant probiotics that have been reported to have the effects of help promote growth and prevent diseases, while most of the other genera have no beneficial or harmful effects on plants. However, 16 genera unique to the soil community and one genus unique to each of the two substrates were not reported to be beneficial or harmful to plants. In addition, among the genera shared by the soil and the two types of substrates, there were only two genera, *Paenibacillus* (Soni et al., 2021) and *Lysobacter* (Laborda et al., 2018) have been reported as plant probiotics, as for the remaining, neither the genera shared by the soil and substrate 1 nor the genera shared by the two types of substrates, were reported to be beneficial or harmful to plants. The results indicated that the tomato root tended to recruit the genus that benefited plant’s growth during its continuous growth in different environments. This study also showed that the genera with higher abundance had significant abundance differences in the three cultivation media, similar to differences observed in bacterial colonies in cucumber roots in previous studies (Zhou et al., 2022). For example, the abundance of some facultative anaerobes in soil (such as *Aquabacteria*, *Acidoborax*, and *Flavobacillium*) (Dahal et al., 2021; Li et al., 2021; Liu L. et al., 2021) was significantly higher than those in the two substrates. Aerobic bacteria (such as *Massilia*, *Della*, and *Asticcacaulis*) (Kim et al., 2014; Du and Yin, 2016; Ishizawa et al., 2019) were significantly lower than those of the two substrates. Such recruitment and colonization of beneficial bacteria in tomatoes are similar to the recruitment of specific functions of intestinal microorganism community, such as the colonization of intestinal-colonized anti-*Beauveria bassiana* activity by *Allium fistulosum* (Xu et al., 2019; Zhou et al., 2019, 2020, 2021; Liu M. et al., 2021), suggesting that the formation of plant/animal flora complex by recruiting beneficial microorganism community is a common phenomenon in nature.

Generally, in greenhouse tomato planting, the seedlings are transplanted from the seedling-raising substrate to the soil for field planting. However, it is still unclear if the root microorganism community has changed and how before and after the transplantation. In this study, the composition changes in root microflora after tomato seedlings were transplanted into the soil from two types of substrates were analyzed. The results showed that regardless of the abundance or diversity of microflora reflected from the *α*-diversity analysis, the bacterial communities after transplantation were closer to the characteristics of soil microflora. In addition, *β*-diversity analysis clustered the transplanting microflora and soil-cultivating bacteria together (no significant difference). These results suggest that the structure of tomato root flora was gradually assimilated with the soil flora structure under the influence of the soil environment (including biological or non-biological) after being transplanted into the soil from the two substrates. In particular, at the genus level, Venn analysis showed that after transplantation, almost all of the soil bacterial community successfully colonized the roots, but a small part of the genera from the substrate bacterial community was lost. Specifically, the plant probiotics of the above 11 genera either from the soil or the substrates were all successfully colonized in the root. *Via* the abundance analysis, the abundance of the top 20 genera after transplantation was consistent with that of the soil flora. Among them, the abundance of three genera (*Devosia*, *Rhizobium*, and *Lysobacter*) was significantly increased, whereas that of *Bradyrhizobium* was significantly decreased after transplantation from substrate 1 to the soil. The abundance of the two genera, *Devosia* and *Bradyrhizobium*, was significantly reduced after their transplantation from substrate 2 to the soil.

Based on the results of this study, it could be concluded that: (1) there was a core bacterial community group in tomato roots, which was not affected by the cultivation medium type or transplantation action, and it preferred to contain almost all plant probiotics endowed by the environment; (2) after transplantation, the structure, and abundance of tomato root community were similar to that in soil; (3) In a certain plant genotype, the reported beneficial bacteria in tomato root are always present, even if the abundance ratio of the whole flora is changed by transplantation. Recently, with the advancement of high-throughput sequencing technology and culturomics technology, research on the construction and interaction of plant microbiota, especially the root microbiota, has become a focal point. The following consensus has been formed: (1) There are plant “core microflora,” which can establish repeatable connections with specific hosts in a wide range of environments (Edwards et al., 2015; Lemanceau et al., 2017; Fitzpatrick et al., 2018; Hamonts et al., 2018; Roman-Reyna et al., 2019; Trivedi et al., 2020); (2) The construction of plant core microbial communities is not random but driven by the complex interaction between microorganisms, plant hosts, and the environment (Carlström et al., 2019). In addition, many genera of the core microbiota of different plant species are widely distributed among different plants. For example, the microbiota of barley, rice, sugarcane, grape, citrus, soybean, and Arabidopsis have similarities at the genus level, and the specific genera include Pseudomonas, Agrobacterium, *Methylobacter*, *Sphingomonas*, *Erwinia*, *Cladosporium*, and *Coniothyrium*, *Resinicium*, *Fusarium* (Trivedi et al., 2020). In this study, 79 bacterial genera in tomatoes in different media overlapped with only 3 genera (*Pseudomonas*, *Methylobacterium*, and *Sphingomonas*) that were mentioned above. Interestingly, in our previous study, we compared and analyzed the bacterial community in the cucumber roots under different cultivation medium conditions in the same greenhouse as in this study, and 26 common genera were obtained; of which 16 genera were the same as the common genera in tomato roots (except for three genera: *Devosia*, *Bradyrhizobium*, and *Pseudomonas*, most of which did not have any reported effects on plants), and four same common families that could not be identified to genus (Zhou et al., 2022). These phenomena suggest that similar growth environments may produce similar core groups; however, most of the bacterial genera that are potentially beneficial to plants are determined to a greater extent by different plant types.

Further, the abundance analysis of metabolic pathways showed that the differences in metabolism-related pathways among the three cultivation media were the largest. The main differential pathways between the two matrices and soil were compared (both upregulated and downregulated by more than two times). The upregulated metabolic pathways were mostly related to the promotion of plant growth; for example, sesquiterpenoid and triterpenoid biosynthesis, biosynthesis of monoterpenoid, and steroid hormone biosynthesis. Most of the downregulated pathways were related to antibiotic synthesis, such as biosynthesis of enediyne antibiotics, flavonoid biosynthesis, stilbenoid, diarylheptanoid, and gingerol biosynthesis, biosynthesis of 12-, 14-, and 16-membered macrolides. This result was similar to the functional difference characteristics of bacteria in cucumber roots in different cultivation media, i.e., the abundance of growth-promoting metabolic pathways (such as flavonoid and sesquiterpenoid substance synthesis) in the matrix was significantly increased^[12]^. After being transplanted to the soil from substrate 1, it still showed that the largest difference existed in metabolic pathways compared with substrate 1 or soil environment. Specifically, the significantly upregulated pathways compared with substrate1 mainly referred to antibiotic synthesis-related pathways (including biosynthesis of 12-, 14-, and 16-membered macrolides) and plant growth promotion-related pathway (biosynthesis of monoterpenoid). It is worth noting that the plant growth promotion-related pathway (biosynthesis of monoterpenoid) was also upregulated compared with the soil. After being transplanted to the soil from substrate 2, the difference in metabolic pathways was also the largest. Specifically, compared with substrate 2, the markedly upregulated antibiotic synthesis-related pathways including biosynthesis of energize antibiosis, etc. Specifically, compared with soil, significantly upregulated the plant growth-promoting pathway including the biosynthesis of monoterpenoid. Regarding flora function prediction, the two substrate environments were more conducive to the growth-promoting potential of the flora, and the flora in the soil was more inclined to play the antibacterial potential. It was speculated that because there were more abundant plant pathogenic microorganisms naturally existing in the soil, the microbial community in the soil environment tended to exhibit antibacterial activity, while in the substrate, it activated growth-promoting characteristics.

To sum up, the results demonstrated that the differences in bacterial species were not significant in the three cultivation media. However, the abundance differences were significant, and a core bacterial community containing most of the plant-beneficial bacteria was observed. After transplantation from the two substrates, the structure and abundance of the bacterial community were more similar to that of the soil. Despite the fact that the abundance ratio of the whole bacterial community was altered by transplantation, the potentially plant-beneficial bacteria in the tomato roots were always present. In addition, from the perspective of bacterial community functional properties, the two substrate environments were more conducive to the growth-promoting potential of the bacterial community, and the bacterial community in the soil was more inclined to play the antibacterial potential. Our findings provide theoretical data support for constructing artificial reconstituted bacteria in greenhouse planting mode. The results of our analysis suggest that the tomato root system stably maintains a core bacterial community containing most of the beneficial bacteria for plants, with functions such as growth promotion and inhibition. In the next step, we plan to employ culturomics approaches to obtain more types of pure cultures from the tomato root system, construct a simplified functional microbial community, and develop stable microbial agents and technological systems that can improve tomato stress resistance and disease resistance.

## Data availability statement

The datasets presented in this study can be found in online repositories. The names of the repository/repositories and accession number(s) can be found at: NCBI BioProject—PRJNA943126.

## Author contributions

XZ was responsible for the conceptualization, supervision, data curation, and writing—reviewing and editing. QL was responsible for conceptualization, methodology, software, data curation, and writing—reviewing and editing. FZ was responsible for visualization and investigation. SF was responsible for investigation. XZ was responsible for investigation. KY was responsible for writing-reviewing and editing. CZ was responsible for visualization. XW was responsible for conceptualization, supervision, software, validation, and writing—original draft preparation. All authors contributed to the article and approved the submitted version.

## Funding

This work was funded by Basic research projects of the SE&I Integration of Qilu University of Technology [grant numbers 2022PYI009, 2022PY016, and 2022PT105], Innovative Team Project of Ji’nan Government [grant number 2021GXRC040], the Key Research and Development Program of Shandong Province [grant number 2021TZXD002], the Shandong Provincial Science and Technology SME Innovation Capability Improving Project [grant number 2022TSGC2060], and the National Natural Science Foundation of China [grant number 31901928].

## Conflict of interest

The authors declare that the research was conducted in the absence of any commercial or financial relationships that could be construed as a potential conflict of interest.

## Publisher’s note

All claims expressed in this article are solely those of the authors and do not necessarily represent those of their affiliated organizations, or those of the publisher, the editors and the reviewers. Any product that may be evaluated in this article, or claim that may be made by its manufacturer, is not guaranteed or endorsed by the publisher.

## Footnotes

^1^ http://www.arb-silva.de

^2^ http://eggnog.embl.de/

^3^ http://www.genome.jp/kegg/
