# Supplementary material for: Effects of different cultivation media on root bacterial community characteristics of greenhouse tomatoes
Source: Front Microbiol. 2023 May 18;14:1182347. doi: 10.3389/fmicb.2023.1182347 (PMC10232838; doi:10.3389/fmicb.2023.1182347)
Supplement: Supplementary file 1 [file Table_1.DOCX]

Supplementary Material

**Effects of different Cultivation Media on Root Bacterial Community Characteristics of Greenhouse Tomatoes**

**Xinjian Zhang ^1^, Qiang Li ^1^, Fangyuan Zhou ^1^, Susu Fan ^1^, Xiaoyan Zhao ^1^, Chi Zhang ^1^, Kun Yan ^2^, Xiaoqing Wu ^1*^**

*** Correspondence:** Xiaoqing Wu: wxq@qlu.edu.cn

# Supplementary Tables

## Table S1. Information of sequencing data^*^.

| **Sample** | **Optimized reads** | **Average length** | **Total bases** | **Q30** | **Q20** | **Primer** |
| --- | --- | --- | --- | --- | --- | --- |
| S-1 | 24791 | 373.7573 | 9265816 | 95.66601 | 98.68897 | 799F_1193R |
| S-2 | 24217 | 375.488 | 9093192 | 95.4017 | 98.6031 | 799F_1193R |
| S-3 | 24774 | 374.5305 | 9278618 | 95.39686 | 98.57629 | 799F_1193R |
| S-4 | 24401 | 374.1882 | 9130567 | 95.43358 | 98.60658 | 799F_1193R |
| S-5 | 23562 | 374.8389 | 8831954 | 95.30646 | 98.55218 | 799F_1193R |
| S-6 | 23767 | 374.9296 | 8910952 | 95.3393 | 98.56014 | 799F_1193R |
| M1-1 | 24671 | 373.9444 | 9225582 | 95.7058 | 98.68817 | 799F_1193R |
| M1-2 | 24912 | 375.8275 | 9362614 | 95.43944 | 98.61171 | 799F_1193R |
| M1-3 | 23044 | 377.1377 | 8690762 | 95.11571 | 98.5058 | 799F_1193R |
| M1-4 | 22018 | 375.447 | 8266591 | 95.62011 | 98.65687 | 799F_1193R |
| M1-5 | 21256 | 377.4513 | 8023105 | 95.5648 | 98.65678 | 799F_1193R |
| M1-6 | 23313 | 375.3374 | 8750240 | 95.64919 | 98.67812 | 799F_1193R |
| M2-1 | 24595 | 376.407 | 9257730 | 95.46619 | 98.64474 | 799F_1193R |
| M2-2 | 23037 | 376.5922 | 8675554 | 95.37535 | 98.55159 | 799F_1193R |
| M2-3 | 24583 | 376.2173 | 9248551 | 95.52487 | 98.65285 | 799F_1193R |
| M2-4 | 23898 | 376.5049 | 8997714 | 95.49946 | 98.62418 | 799F_1193R |
| M2-5 | 24082 | 376.3078 | 9062244 | 95.43295 | 98.61769 | 799F_1193R |
| M2-6 | 23236 | 376.202 | 8741430 | 95.27131 | 98.55019 | 799F_1193R |
| M1_to_S-1 | 14351 | 374.6605 | 5376753 | 95.84429 | 98.7576 | 799F_1193R |
| M1_to_S-2 | 23656 | 374.3846 | 8856442 | 95.69903 | 98.70712 | 799F_1193R |
| M1_to_S-3 | 23102 | 374.303 | 8647149 | 95.71882 | 98.69328 | 799F_1193R |
| M1_to_S-4 | 21947 | 375.0757 | 8231787 | 95.52928 | 98.63042 | 799F_1193R |
| M1_to_S-5 | 23354 | 374.5101 | 8746309 | 95.55956 | 98.63049 | 799F_1193R |
| M1_to_S-6 | 23204 | 375.5803 | 8714965 | 95.5824 | 98.64343 | 799F_1193R |
| M2_to_S-1 | 24997 | 375.9656 | 9398013 | 95.62536 | 98.67309 | 799F_1193R |
| M2_to_S-2 | 23035 | 375.1816 | 8642308 | 95.63907 | 98.6721 | 799F_1193R |
| M2_to_S-3 | 23875 | 374.4775 | 8940651 | 95.46161 | 98.61053 | 799F_1193R |
| M2_to_S-4 | 23786 | 376.4642 | 8954578 | 95.53904 | 98.62708 | 799F_1193R |
| M2_to_S-5 | 25000 | 375.5592 | 9388980 | 95.62616 | 98.67071 | 799F_1193R |
| M2_to_S-6 | 24312 | 374.5801 | 9106792 | 95.897 | 98.73704 | 799F_1193R |

^*^The number of raw reads is 702,776*2 (duplicate the single-end sequences for both ends), the total raw base number is 423,071,152; the number of optimized reads is 702,776, the total optimized base number is 263,817,943.

**Table S2. List of bacteria that were common/exclusive in comparision among S, M1 and M2 group.**

| **Group^**^**  **Species^*^**  **No.** | **S & M1 & M2** | **S & M1** | **S & M2** | **M1 & M2** | **S** | **M1** | **M2** |
| --- | --- | --- | --- | --- | --- | --- | --- |
|  | *Massilia* | *Mizugakiibacter* | unclassified_f__Rhodanobacteraceae | norank_f__Amb-16S-1323 | *Permianibacter* | *Actinocatenispora* | *Paludibaculum* |
|  | *Roseomonas* | unclassified_c__Bacteroidia | *Sphingobium* | norank_f__Caulobacteraceae | unclassified_o__Burkholderiales |  |  |
|  | *Devosia* | *Actinoplanes* | *Rhizobacter* | *Chujaibacter* | norank_f__Ardenticatenaceae |  |  |
|  | *Pseudonocardia* | norank_f__Roseiflexaceae | norank_f__Microscillaceae | *Streptacidiphilus* | *Oscillochloris* |  |  |
|  | *Bacillus* | unclassified_f__Myxococcaceae | *Deinococcus* | norank_f__Micropepsaceae | *Methylotenera* |  |  |
|  | *Sphingomonas* | TM7a | *Azohydromonas* | *Aneurinibacillus* | *Lechevalieria* |  |  |
|  | norank_f__Xanthobacteraceae |  | Subgroup_10 | *Castellaniella* | FFCH7168 |  |  |
|  | unclassified_f__Xanthobacteraceae |  | *Bdellovibrio* | *Gemmatimonas* | *Ahniella* |  |  |
|  | unclassified_f__Polyangiaceae |  | *Paenibacillus* | *Dokdonella* | *Agromyces* |  |  |
|  | *Nocardioides* |  | norank_f__norank_o__R7C24 | *Solimonas* | *Kineosporia* |  |  |
|  | *Rhodoplanes* |  | *Microbacterium* | *Bauldia* | *Rhodobacter* |  |  |
|  | *Pseudolabrys* |  | *Cellvibrio* | *Hephaestia* | unclassified_c__Alphaproteobacteria |  |  |
|  | *Pseudaminobacter* |  | *Turneriella* | *Bryobacter* | unclassified_o__Frankiales |  |  |
|  | *Dongia* |  | *Lysobacter* | *Acidothermus* | *Herpetosiphon* |  |  |
|  | *Actinophytocola* |  |  | *Tistrella* | *Ohtaekwangia* |  |  |
|  | unclassified_p__Proteobacteria |  |  | *Granulicella* | *Haliangium* |  |  |
|  | *Marmoricola* |  |  | *Acidisphaera* |  |  |  |
|  | *Sphingopyxis* |  |  |  |  |  |  |
|  | norank_f__norank_o__Xanthomonadales |  |  |  |  |  |  |
|  | *Hyphomicrobium* |  |  |  |  |  |  |
|  | *Dyella* |  |  |  |  |  |  |
|  | *Ramlibacter* |  |  |  |  |  |  |
|  | unclassified_f__Comamonadaceae |  |  |  |  |  |  |
|  | *Caulobacter* |  |  |  |  |  |  |
|  | *Asticcacaulis* |  |  |  |  |  |  |
|  | *Pseudorhodoplanes* |  |  |  |  |  |  |
|  | *Reyranella* |  |  |  |  |  |  |
|  | *Ralstonia* |  |  |  |  |  |  |
|  | *Amycolatopsis* |  |  |  |  |  |  |
|  | *Allorhizobium-Neorhizobium-Pararhizobium-Rhizobium* |  |  |  |  |  |  |
|  | *Arenimonas* |  |  |  |  |  |  |
|  | unclassified_f__Micromonosporaceae |  |  |  |  |  |  |
|  | norank_f__Mitochondria |  |  |  |  |  |  |
|  | norank_f__Hyphomicrobiaceae |  |  |  |  |  |  |
|  | *Mesorhizobium* |  |  |  |  |  |  |
|  | *Bradyrhizobium* |  |  |  |  |  |  |
|  | *Noviherbaspirillum* |  |  |  |  |  |  |
|  | norank_f__Rhodanobacteraceae |  |  |  |  |  |  |
|  | *Ellin6067* |  |  |  |  |  |  |
|  | norank_f__Pedosphaeraceae |  |  |  |  |  |  |
|  | *Methylobacillus* |  |  |  |  |  |  |
|  | *Steroidobacter* |  |  |  |  |  |  |
|  | unclassified_f__Pedosphaeraceae |  |  |  |  |  |  |
|  | *Limnobacter* |  |  |  |  |  |  |
|  | unclassified_f__Xanthomonadaceae |  |  |  |  |  |  |
|  | *Methyloversatilis* |  |  |  |  |  |  |
|  | *Phenylobacterium* |  |  |  |  |  |  |
|  | unclassified_f__Intrasporangiaceae |  |  |  |  |  |  |
|  | *Novosphingobium* |  |  |  |  |  |  |
|  | unclassified_f__Oxalobacteraceae |  |  |  |  |  |  |
|  | Polyangium_brachysporum_group |  |  |  |  |  |  |
|  | unclassified_f__Rhizobiaceae |  |  |  |  |  |  |
|  | *Ensifer* |  |  |  |  |  |  |
|  | *Acidovorax* |  |  |  |  |  |  |
|  | norank_f__Gemmatimonadaceae |  |  |  |  |  |  |
|  | *Streptomyces* |  |  |  |  |  |  |
|  | *Pseudomonas* |  |  |  |  |  |  |
|  | norank_f__Sphingomonadaceae |  |  |  |  |  |  |
|  | *Bosea* |  |  |  |  |  |  |
|  | *Brevundimonas* |  |  |  |  |  |  |
|  | unclassified_f__Sphingomonadaceae |  |  |  |  |  |  |
|  | *Peredibacter* |  |  |  |  |  |  |
|  | norank_f__Methyloligellaceae |  |  |  |  |  |  |
|  | *Kribbella* |  |  |  |  |  |  |
|  | *Pelomonas* |  |  |  |  |  |  |
|  | unclassified_f__Microbacteriaceae |  |  |  |  |  |  |
|  | *Flavobacterium* |  |  |  |  |  |  |
|  | *Ideonella* |  |  |  |  |  |  |
|  | *Altererythrobacter* |  |  |  |  |  |  |
|  | *Ferrovibrio* |  |  |  |  |  |  |
|  | *Acidibacter* |  |  |  |  |  |  |
|  | unclassified_f__Methylophilaceae |  |  |  |  |  |  |
|  | *Pseudarthrobacter* |  |  |  |  |  |  |
|  | *Rubrivivax* |  |  |  |  |  |  |
|  | *Aeromicrobium* |  |  |  |  |  |  |
|  | *Burkholderia-Caballeronia-Paraburkholderia* |  |  |  |  |  |  |
|  | *Mycobacterium* |  |  |  |  |  |  |
|  | unclassified_f__Devosiaceae |  |  |  |  |  |  |
|  | *Bordetella* |  |  |  |  |  |  |

^*^ includes identified genus names, families for bacteria that cannot be identified at the genus level, and other unnamed bacteria.

^**^ S & M1 & M2 represents common bacteria among the three treatments; S & M1 represents common exclusively bacteria among S and M1; S & M2 represents common exclusively bacteria among S and M2; M1 & M2 represents common exclusively bacteria among M1 and M2; S or M1 or M2 represents exclusive bacteria in each treatment, respectively.

**Table S3. List of bacteria that were common/exclusive in comparision among S, M1 and M1_to_S group.**

| **Group^**^**  **Species^*^**  **No.** | **S & M1 & M1_to_S** | **S & M1** | **S & M1_to_S** | **M1 & M1_to_S** | **S** | **M1** | **M1_to_S** |
| --- | --- | --- | --- | --- | --- | --- | --- |
|  | *Massilia* | -null- | *Permianibacter* | norank_f__Micropepsaceae | -null- | norank_f__Amb-16S-1323 | -null- |
|  | *Mizugakiibacter* |  | unclassified_o__Burkholderiales | *Aneurinibacillus* |  | norank_f__Caulobacteraceae |  |
|  | *Roseomonas* |  | unclassified_f__Rhodanobacteraceae | *Castellaniella* |  | *Chujaibacter* |  |
|  | *Devosia* |  | *Sphingobium* | *Gemmatimonas* |  | *Streptacidiphilus* |  |
|  | *Pseudonocardia* |  | norank_f__Ardenticatenaceae | *Dokdonella* |  | *Bauldia* |  |
|  | *Bacillus* |  | *Oscillochloris* | *Solimonas* |  | *Bryobacter* |  |
|  | unclassified_c__Bacteroidia |  | *Methylotenera* | *Hephaestia* |  | *Acidothermus* |  |
|  | *Sphingomonas* |  | *Rhizobacter* | *Actinocatenispora* |  | *Tistrella* |  |
|  | norank_f__Xanthobacteraceae |  | *Lechevalieria* | *Acidisphaera* |  | *Granulicella* |  |
|  | unclassified_f__Xanthobacteraceae |  | norank_f__Microscillaceae |  |  |  |  |
|  | unclassified_f__Polyangiaceae |  | FFCH7168 |  |  |  |  |
|  | *Nocardioides* |  | *Deinococcus* |  |  |  |  |
|  | *Rhodoplanes* |  | *Azohydromonas* |  |  |  |  |
|  | *Pseudolabrys* |  | *Ahniella* |  |  |  |  |
|  | *Pseudaminobacter* |  | Subgroup_10 |  |  |  |  |
|  | *Dongia* |  | *Agromyces* |  |  |  |  |
|  | *Actinophytocola* |  | *Kineosporia* |  |  |  |  |
|  | unclassified_p__Proteobacteria |  | *Rhodobacter* |  |  |  |  |
|  | *Marmoricola* |  | *Bdellovibrio* |  |  |  |  |
|  | *Sphingopyxis* |  | unclassified_c__Alphaproteobacteria |  |  |  |  |
|  | norank_f__norank_o__Xanthomonadales |  | *Paenibacillus* |  |  |  |  |
|  | *Hyphomicrobium* |  | unclassified_o__Frankiales |  |  |  |  |
|  | *Dyella* |  | norank_f__norank_o__R7C24 |  |  |  |  |
|  | *Ramlibacter* |  | *Microbacterium* |  |  |  |  |
|  | *Actinoplanes* |  | *Cellvibrio* |  |  |  |  |
|  | unclassified_f__Comamonadaceae |  | *Turneriella* |  |  |  |  |
|  | *Caulobacter* |  | *Herpetosiphon* |  |  |  |  |
|  | *Asticcacaulis* |  | *Lysobacter* |  |  |  |  |
|  | *Pseudorhodoplanes* |  | *Ohtaekwangia* |  |  |  |  |
|  | *Reyranella* |  | *Haliangium* |  |  |  |  |
|  | *Ralstonia* |  |  |  |  |  |  |
|  | *Amycolatopsis* |  |  |  |  |  |  |
|  | *Allorhizobium-Neorhizobium-Pararhizobium-Rhizobium* |  |  |  |  |  |  |
|  | *Arenimonas* |  |  |  |  |  |  |
|  | norank_f__Roseiflexaceae |  |  |  |  |  |  |
|  | unclassified_f__Micromonosporaceae |  |  |  |  |  |  |
|  | norank_f__Mitochondria |  |  |  |  |  |  |
|  | norank_f__Hyphomicrobiaceae |  |  |  |  |  |  |
|  | *Mesorhizobium* |  |  |  |  |  |  |
|  | *Bradyrhizobium* |  |  |  |  |  |  |
|  | unclassified_f__Myxococcaceae |  |  |  |  |  |  |
|  | *Noviherbaspirillum* |  |  |  |  |  |  |
|  | norank_f__Rhodanobacteraceae |  |  |  |  |  |  |
|  | Ellin6067 |  |  |  |  |  |  |
|  | norank_f__Pedosphaeraceae |  |  |  |  |  |  |
|  | *Methylobacillus* |  |  |  |  |  |  |
|  | *Steroidobacter* |  |  |  |  |  |  |
|  | unclassified_f__Pedosphaeraceae |  |  |  |  |  |  |
|  | *Limnobacter* |  |  |  |  |  |  |
|  | unclassified_f__Xanthomonadaceae |  |  |  |  |  |  |
|  | *Methyloversatilis* |  |  |  |  |  |  |
|  | *Phenylobacterium* |  |  |  |  |  |  |
|  | unclassified_f__Intrasporangiaceae |  |  |  |  |  |  |
|  | *Novosphingobium* |  |  |  |  |  |  |
|  | unclassified_f__Oxalobacteraceae |  |  |  |  |  |  |
|  | Polyangium_brachysporum_group |  |  |  |  |  |  |
|  | unclassified_f__Rhizobiaceae |  |  |  |  |  |  |
|  | *Ensifer* |  |  |  |  |  |  |
|  | *Acidovorax* |  |  |  |  |  |  |
|  | norank_f__Gemmatimonadaceae |  |  |  |  |  |  |
|  | *Streptomyces* |  |  |  |  |  |  |
|  | *Pseudomonas* |  |  |  |  |  |  |
|  | norank_f__Sphingomonadaceae |  |  |  |  |  |  |
|  | *Bosea* |  |  |  |  |  |  |
|  | TM7a |  |  |  |  |  |  |
|  | *Brevundimonas* |  |  |  |  |  |  |
|  | unclassified_f__Sphingomonadaceae |  |  |  |  |  |  |
|  | *Peredibacter* |  |  |  |  |  |  |
|  | norank_f__Methyloligellaceae |  |  |  |  |  |  |
|  | *Kribbella* |  |  |  |  |  |  |
|  | *Pelomonas* |  |  |  |  |  |  |
|  | unclassified_f__Microbacteriaceae |  |  |  |  |  |  |
|  | *Flavobacterium* |  |  |  |  |  |  |
|  | *Ideonella* |  |  |  |  |  |  |
|  | *Altererythrobacter* |  |  |  |  |  |  |
|  | *Ferrovibrio* |  |  |  |  |  |  |
|  | *Acidibacter* |  |  |  |  |  |  |
|  | unclassified_f__Methylophilaceae |  |  |  |  |  |  |
|  | *Pseudarthrobacter* |  |  |  |  |  |  |
|  | *Rubrivivax* |  |  |  |  |  |  |
|  | *Aeromicrobium* |  |  |  |  |  |  |
|  | *Burkholderia-Caballeronia-Paraburkholderia* |  |  |  |  |  |  |
|  | *Mycobacterium* |  |  |  |  |  |  |
|  | unclassified_f__Devosiaceae |  |  |  |  |  |  |
|  | *Bordetella* |  |  |  |  |  |  |

^*^ includes identified genus names, families for bacteria that cannot be identified at the genus level, and other unnamed bacteria.

^**^ S & M1 & M1_to_S represents common bacteria among the three treatments; S & M1 represents common exclusively bacteria among S and M1; S & M1_to_S represents common exclusively bacteria among S and M1_to_S; M1 & M1_to_S represents common exclusively bacteria among M1 and M1_to_S; S or M1 or M1_to_S represents exclusive bacteria in each treatment, respectively.

**Table S4. List of bacteria that were common/exclusive in comparision among S, M2 and M2_to_S group.**

| **Group^**^**  **Species^*^**  **No.** | **S & M2 & M2_to_S** | **S & M2** | **S & M2_to_S** | **M2 & M2_to_S** | **S** | **M2** | **M2_to_S** |
| --- | --- | --- | --- | --- | --- | --- | --- |
|  | unclassified_f__Rhodanobacteraceae | norank_f__norank_o__R7C24 | *Permianibacter* | norank_f__Amb-16S-1323 | unclassified_c__Bacteroidia | norank_f__Caulobacteraceae | -null- |
|  | *Massilia* |  | unclassified_o__Burkholderiales | norank_f__Micropepsaceae |  | *Chujaibacter* |  |
|  | *Roseomonas* |  | *Mizugakiibacter* | *Aneurinibacillus* |  | *Streptacidiphilus* |  |
|  | *Sphingobium* |  | norank_f__Ardenticatenaceae | *Castellaniella* |  | *Gemmatimonas* |  |
|  | *Devosia* |  | *Oscillochloris* | *Acidothermus* |  | *Dokdonella* |  |
|  | *Pseudonocardia* |  | *Methylotenera* |  |  | *Solimonas* |  |
|  | *Bacillus* |  | *Lechevalieria* |  |  | *Bauldia* |  |
|  | *Sphingomonas* |  | FFCH7168 |  |  | *Hephaestia* |  |
|  | norank_f__Xanthobacteraceae |  | *Actinoplanes* |  |  | *Bryobacter* |  |
|  | unclassified_f__Xanthobacteraceae |  | *Ahniella* |  |  | *Paludibaculum* |  |
|  | unclassified_f__Polyangiaceae |  | norank_f__Roseiflexaceae |  |  | *Tistrella* |  |
|  | *Nocardioides* |  | *Agromyces* |  |  | *Granulicella* |  |
|  | *Rhodoplanes* |  | *Kineosporia* |  |  | *Acidisphaera* |  |
|  | *Pseudolabrys* |  | unclassified_f__Myxococcaceae |  |  | norank_f__Caulobacteraceae |  |
|  | *Pseudaminobacter* |  | *Rhodobacter* |  |  |  |  |
|  | *Dongia* |  | unclassified_c__Alphaproteobacteria |  |  |  |  |
|  | *Actinophytocola* |  | unclassified_o__Frankiales |  |  |  |  |
|  | unclassified_p__Proteobacteria |  | TM7a |  |  |  |  |
|  | *Rhizobacter* |  | *Herpetosiphon* |  |  |  |  |
|  | *Marmoricola* |  | *Ohtaekwangia* |  |  |  |  |
|  | *Sphingopyxis* |  | *Haliangium* |  |  |  |  |
|  | norank_f__norank_o__Xanthomonadales |  |  |  |  |  |  |
|  | *Hyphomicrobium* |  |  |  |  |  |  |
|  | norank_f__Microscillaceae |  |  |  |  |  |  |
|  | *Dyella* |  |  |  |  |  |  |
|  | *Ramlibacter* |  |  |  |  |  |  |
|  | *Deinococcus* |  |  |  |  |  |  |
|  | *Azohydromonas* |  |  |  |  |  |  |
|  | unclassified_f__Comamonadaceae |  |  |  |  |  |  |
|  | *Caulobacter* |  |  |  |  |  |  |
|  | *Asticcacaulis* |  |  |  |  |  |  |
|  | *Pseudorhodoplanes* |  |  |  |  |  |  |
|  | *Reyranella* |  |  |  |  |  |  |
|  | *Ralstonia* |  |  |  |  |  |  |
|  | *Amycolatopsis* |  |  |  |  |  |  |
|  | *Allorhizobium-Neorhizobium-Pararhizobium-Rhizobium* |  |  |  |  |  |  |
|  | *Arenimonas* |  |  |  |  |  |  |
|  | Subgroup_10 |  |  |  |  |  |  |
|  | unclassified_f__Micromonosporaceae |  |  |  |  |  |  |
|  | norank_f__Mitochondria |  |  |  |  |  |  |
|  | norank_f__Hyphomicrobiaceae |  |  |  |  |  |  |
|  | *Mesorhizobium* |  |  |  |  |  |  |
|  | *Bradyrhizobium* |  |  |  |  |  |  |
|  | *Noviherbaspirillum* |  |  |  |  |  |  |
|  | norank_f__Rhodanobacteraceae |  |  |  |  |  |  |
|  | *Bdellovibrio* |  |  |  |  |  |  |
|  | Ellin6067 |  |  |  |  |  |  |
|  | norank_f__Pedosphaeraceae |  |  |  |  |  |  |
|  | *Methylobacillus* |  |  |  |  |  |  |
|  | *Steroidobacter* |  |  |  |  |  |  |
|  | unclassified_f__Pedosphaeraceae |  |  |  |  |  |  |
|  | *Limnobacter* |  |  |  |  |  |  |
|  | unclassified_f__Xanthomonadaceae |  |  |  |  |  |  |
|  | *Methyloversatilis* |  |  |  |  |  |  |
|  | *Paenibacillus* |  |  |  |  |  |  |
|  | *Phenylobacterium* |  |  |  |  |  |  |
|  | unclassified_f__Intrasporangiaceae |  |  |  |  |  |  |
|  | *Microbacterium* |  |  |  |  |  |  |
|  | *Novosphingobium* |  |  |  |  |  |  |
|  | unclassified_f__Oxalobacteraceae |  |  |  |  |  |  |
|  | Polyangium_brachysporum_group |  |  |  |  |  |  |
|  | unclassified_f__Rhizobiaceae |  |  |  |  |  |  |
|  | *Ensifer* |  |  |  |  |  |  |
|  | *Acidovorax* |  |  |  |  |  |  |
|  | norank_f__Gemmatimonadaceae |  |  |  |  |  |  |
|  | *Streptomyces* |  |  |  |  |  |  |
|  | *Cellvibrio* |  |  |  |  |  |  |
|  | *Pseudomonas* |  |  |  |  |  |  |
|  | norank_f__Sphingomonadaceae |  |  |  |  |  |  |
|  | *Bosea* |  |  |  |  |  |  |
|  | *Turneriella* |  |  |  |  |  |  |
|  | *Brevundimonas* |  |  |  |  |  |  |
|  | unclassified_f__Sphingomonadaceae |  |  |  |  |  |  |
|  | *Peredibacter* |  |  |  |  |  |  |
|  | norank_f__Methyloligellaceae |  |  |  |  |  |  |
|  | *Kribbella* |  |  |  |  |  |  |
|  | *Pelomonas* |  |  |  |  |  |  |
|  | unclassified_f__Microbacteriaceae |  |  |  |  |  |  |
|  | *Flavobacterium* |  |  |  |  |  |  |
|  | *Ideonella* |  |  |  |  |  |  |
|  | *Altererythrobacter* |  |  |  |  |  |  |
|  | *Ferrovibrio* |  |  |  |  |  |  |
|  | *Acidibacter* |  |  |  |  |  |  |
|  | *Lysobacter* |  |  |  |  |  |  |
|  | unclassified_f__Methylophilaceae |  |  |  |  |  |  |
|  | *Pseudarthrobacter* |  |  |  |  |  |  |
|  | *Rubrivivax* |  |  |  |  |  |  |
|  | *Aeromicrobium* |  |  |  |  |  |  |
|  | *Burkholderia*-*Caballeronia*-*Paraburkholderia* |  |  |  |  |  |  |
|  | *Mycobacterium* |  |  |  |  |  |  |
|  | unclassified_f__Devosiaceae |  |  |  |  |  |  |
|  | *Bordetella* |  |  |  |  |  |  |
|  | unclassified_f__Rhodanobacteraceae |  |  |  |  |  |  |

^*^ includes identified genus names, families for bacteria that cannot be identified at the genus level, and other unnamed bacteria.

^**^ S & M2 & M2_to_S represents common bacteria among the three treatments; S & M2 represents common exclusively bacteria among S and M2; S & M2_to_S represents common exclusively bacteria among S and M2_to_S; M2 & M2_to_S represents common exclusively bacteria among M2 and M2_to_S; S or M2 or M2_to_S represents exclusive bacteria in each treatment, respectively.
